# Photoinduced radical emission from flexible organic crystals

**DOI:** 10.1038/s41377-026-02208-6

**Published:** 2026-05-19

**Authors:** Xuan Zhang, Wenyuan Pan, Yuqi Tang, Ting Huang, Xuesong Yang, Hui Mao, Feiying Ruan, Qing Luo, Lin Li, Hongyu Zhang, Yujian Zhang, Quan Li

**Affiliations:** 1 Key Laboratory of the Ministry of Education for Advanced Catalysis Materials, Department of Chemistry, Zhejiang Normal University, Yingbin Road No. 688, Jinhua, 321004 China; 2 Institute of Advanced Materials, School of Chemistry and Chemical Engineering, and School of Electronic Science & Engineering, Southeast University, Nanjing, 211189 China; 3 State Key Laboratory of Supramolecular Structure and Materials, College of Chemistry, Jilin University, Qianjin Street No. 2699, Changchun, 130012 China; 4 Materials Science Graduate Program, Kent State University, Kent, OH 44242 USA

**Keywords:** Optical materials and structures, Optical physics

## Abstract

Organic single crystals (OSCs) exhibiting excellent flexibility and unique optical properties are particularly promising for applications in optical/optoelectronic devices and sensors. Nevertheless, the fabrication of flexible OSCs with radical luminescence remains a major challenge, as most radicals are non-emissive in the condensed state. Here, we propose a photoactivated radical self-doping strategy to simultaneously achieve radical emission and flexibility in OSCs. By simple UV irradiation in air, the nearly non-emissive crystalline naphthyl benzoate derivative (**NPBr**) produces intense blue fluorescence with a high solid-state photoluminescence quantum yield (PLQY) of 47.7%, representing a remarkable 60-fold enhancement from its initial value of 0.8%. The combined experimental and theoretical analyses reveal that the observed luminescence arises from trace oxygen-centered radical species, which are generated via the photodissociation of **NPBr** and stabilized by the spatial confinement of the crystalline matrix. Moreover, the synergistic effects of hydrogen bonding, halogen interactions, and π-π stacking endow the resulting crystal with high elasticity and bendability under external stress, as evidenced by Young’s modulus of ~9.76 GPa. Notably, this photodissociation process achieves two objectives concurrently: yielding stable oxygen radicals and preserving the intrinsic crystal flexibility, thereby enabling the crystal to function as a flexible optical waveguide with low loss coefficients of 0.584 and 0.806 dB mm^−1^ for the straight and bent states, respectively. This proposed strategy is conceptually innovative and operationally straightforward, offering a universal route for designing flexible OSCs with radical luminescence.

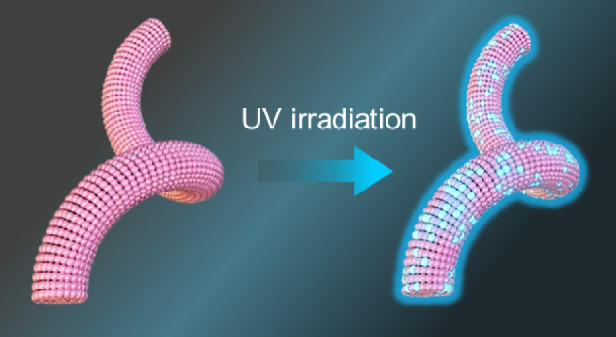

## Abstract (web summary)

Photoinduced radical doping strategy enables blue luminescence and elastic bending (>180°) in organic crystals. Confined oxygen radicals and dynamic π-π/Br···O interactions enable flexibility with low optical loss, advancing flexible optoelectronics.

## Introduction

**Fig. 1:**
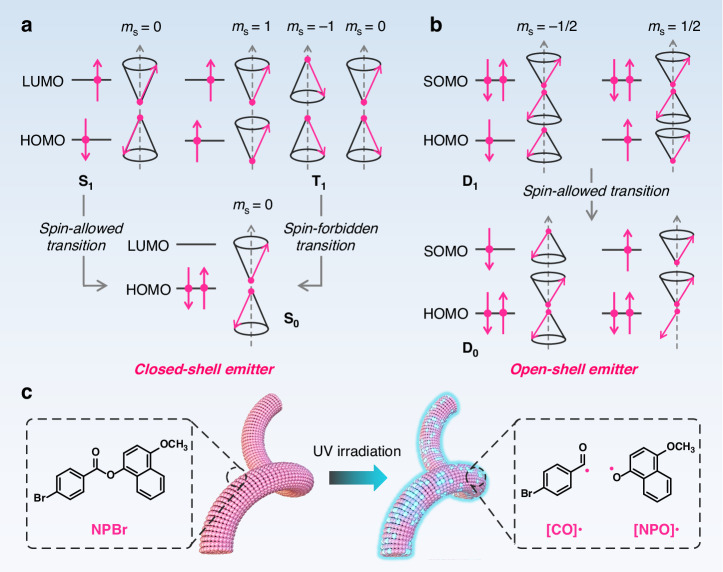
Design and preparation of radical luminescent flexible organic single crystals. **a**, **b** Schematic diagrams of excited-state spin configurations for closed-shell and open-shell emissive molecules, with *m*_s_ denoting the projection of the total spin angular momentum onto the external magnetic field direction. **c** Chemical structures of **NPBr** and open-shell radicals [CO]• and [NPO]•

Flexible optoelectronics have emerged as a transformative technology, attracting considerable attention for their potential to advance and revolutionize human life^1^. With advantages such as lightweight, compliance, and mechanical deformability, these devices unlock unprecedented opportunities in flexible display technologies, wearable health monitors, implantable medical diagnostics, artificial intelligence, and soft robotics. Organic single crystals (OSCs) are considered a promising material platform for next-generation high-performance optoelectronics^2^, offering a unique combination of superior photoelectric characteristics—arising from their reduced grain boundaries and defects—along with intrinsic benefits in molecular diversity, low-cost, and solution processability. But the inherent high brittleness of OSCs limits their fabrication and integration with other device components. Recent studies demonstrate that organic crystals with sufficiently high aspect ratios exhibit remarkable flexibility, enabling elastic or plastic deformation^3–6^. Although the mechanistic underpinnings of this behavior have been and continue to be subjects of ongoing debate, its application potential remains unequivocal. Indeed, the long-range ordered lattices of these molecules show promise in diverse fields, including muscle-mimetic biomaterials, optical devices, and artificial mechanosensors^7–10^. To achieve their use in optical and electronic devices, developing OSCs that combine flexibility with optical/optoelectronic properties is critical. The current approach predominantly employs closed-shell molecules, where electrons are paired, to achieve flexible OSCs (Fig. 1a)^11–15^. For instance, Takagi et al. reported a highly flexible pentafluorophenyl-derived crystal with fluorescence and exceptional optical waveguide performance^16^. Similarly, Zhang and co-workers prepared an elastic Schiff base crystal, emitting bright orange-red fluorescence under mechanical stress^17^. Chandrasekar et al. reported two distinct polymorphs of a triphenylamine derivative, characterized by flexible structures and exhibiting green and orange emissions. These polymorphs demonstrated thermally activated delayed fluorescence and room-temperature phosphorescence, respectively^15^. In our group, a host–guest doping strategy was introduced to concurrently achieve room-temperature phosphorescence and flexibility in OSCs^18^. Notably, Li et al. demonstrated persistent phosphorescence in flexible organic crystals, attributing deformability under external force to alkoxy chain-mediated structural adaptability^19^.

Organic radical materials, characterized as open-shell molecules, generate doublet excited states and exhibit spin-allowed fluorescence upon photoexcitation, arising from the lowest doublet excited state (D_1_; Fig. 1b)^20–22^. Unlike closed-shell molecules, the fluorescent processes of radicals are not subject to annihilation by the triplet state, for instance, singlet–triplet intersystem crossing. These unique luminescence properties may enable flexible OSCs to advance applications in optical sensing, security imaging, and scintillator technologies^23–25^. Although a rare example of a plastically deformable OSC derived from a stable dithiadiazolyl radical has been reported by Naumov et al.^26^, there are no reports on the luminescent flexible OSCs featuring radical emission, primarily due to two challenges. First, generating radical emissions under ambient conditions remains challenging due to the inherent instability of radical species in air. By employing rational molecular design strategies such as spin delocalization and steric hindrance, a series of stable luminescent radicals, primarily based on triarylmethyl radicals, have been developed^20–25,27,28^. However, such radicals often exhibit aggregation-caused quenching in the condensed state, primarily resulting from electron transfer and spin-exchange interactions. Although efficient solid-state radical emission can be achieved through physical doping and steric control strategies^29–33^, these luminescent radical systems, such as triarylmethyl derivatives, are generally not amenable to the fabrication of flexible OSCs due to their highly twisted propeller structures. These radicals suffer from severely diminished photoluminescence quantum yield (PLQY) in the crystalline state and are frequently non-emissive. Second, the correlation between the structural features of stable radical molecules with luminescence and the mechanical deformability of crystals remains unclear.

Herein, we report the first instance of radical luminescence in the flexible OSCs via an ingenious photoactivated radical self-doping strategy. Needle-like OSCs were prepared using a planar benzoate ester derivative, **NPBr** (Fig. 1c), which is nearly non-emissive with a PLQY of 0.8%. Impressively, the photoinduced luminescence has been clearly observed under UV irradiation. After 10 min of irradiation, **NPBr** crystals emit bright blue luminescence with a PLQY of up to 47.7%. Experimental analysis utilizing single-crystal X-ray diffraction (XRD), liquid chromatography, and electron paramagnetic resonance (EPR) has verified that the emergent photoluminescence (PL) peak at 480 nm is attributed to the radical luminescence of oxygen-centered radical species ([NPO]•). Moreover, centimeter-scale acicular OSCs of **NPBr** demonstrate reversible mechanical flexibility. Of particular note, these flexible crystals, which exhibit radical luminescence, were successfully applied in optical waveguides.

## Results

### Molecular design and photoinduced luminescence properties

**Fig. 2:**
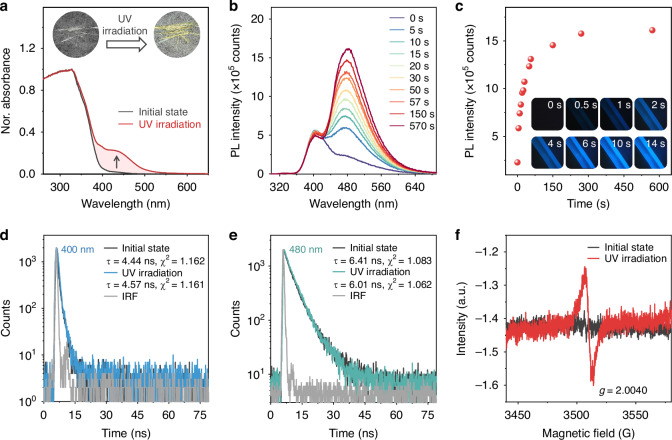
Photoinduced luminescence property of NPBr. **a** Absorption spectra of **NPBr** crystalline powders before and after UV irradiation, with insets showing the corresponding photographs of the crystals under ambient light. **b** PL spectra and **c** integrated PL intensity at 480 nm of **NPBr** crystalline powders as a function of UV irradiation time (0–10 min), with insets in (**c**) showing corresponding PL photographs of a single **NPBr** crystal. A narrowband filter (380 nm center wavelength, 10 nm bandwidth) and dichroic mirrors were installed in front of the camera lens for imaging. **d**, **e** Time-resolved PL decay curves of **NPBr** (*λ*_em_ = 400 nm and 480 nm) before and after UV irradiation. **f** EPR spectra of **NPBr** crystalline powders before and after UV irradiation

The organic dye **NPBr**, a derivative of naphthyl benzoate, was prepared by a one-step esterification reaction in a high yield of 76%. The synthesis followed well-established procedures^6,34^ and is detailed in the Supporting Information. Numerous needle-shaped crystals of **NPBr** were obtained by recrystallization from a mixture of dichloromethane and methanol at room temperature. The resulting crystals appeared colorless and were nearly non-emissive under UV irradiation. As illustrated in Fig. 2a, the ultraviolet-visible (UV-vis) absorption spectrum of **NPBr** displayed a broad absorption band in the range of 260–402 nm, consistent with its colorless appearance. Time-dependent density functional theory (TD-DFT) calculations indicated that the absorption peak at 325 nm was attributed to π–π* transitions of naphthyl benzoate (Fig. S1). Upon photoirradiation for several seconds, the colorless crystals gradually transformed to yellow. A new absorption peak emerged at 441 nm, indicating the generation of new emissive species with a narrow optical gap. The pristine **NPBr** crystals showed a distinct PL peak at 400 nm, accompanied by a weak, broad shoulder band at 480 nm (Fig. 2b). The shoulder arises from the unavoidable light irradiation of **NPBr** by the excitation source of the spectrometer during measurement. Interestingly, when the **NPBr** crystalline powders were continuously irradiated with an additional UV lamp at room temperature in air, the PL intensity of the shoulder peak gradually increased. If this experiment were conducted in the dark, a bright blue luminescence would be observed in insets of Fig. 2c. These results demonstrated that **NPBr** crystals exhibited a photoinduced luminescence behavior. Moreover, the intensity of this photoinduced luminescent peak increased sharply (Fig. 2c) as the irradiative time within 75 s. When the irradiation time was extended from 75 s to 10 min, the rate of increase in PL intensity slowed. After 10 min of photoirradiation, **NPBr** crystals achieved maximum PL intensity with PLQY of 47.7%, exhibiting a highly competitive PLQY value among reported crystalline luminescent radicals^31–33,35–37^. As depicted in Fig. S2, the excitation spectra of photoirradiated **NPBr** powders correlate well with their absorption spectra, revealing additional long-wavelength absorption features associated with emission processes. Interestingly, the photoinduced luminescence behavior of **NPBr** was also observed in CH_2_Cl_2_ and polymethyl methacrylate (PMMA) films (Fig. S3). As depicted in Fig. 2d, e, the PL lifetime of this dominant peak at 480 nm was measured and calculated to be 6.01 ns, indicative of its fluorescence characteristics^38–40^. Together with the PLQY values, the radiative (*k*_r_) and non-radiative (*k*_nr_) decay rate constants were determined to be 7.9 × 10^7^ s^−1^ and 8.7 × 10^7^ s^−1^, respectively. The *k*_r_ value was relatively high compared to those of other emitting π-radicals. Notably, the new generation of emitting species was highly sensitive to photoirradiation. Thus, during PL measurements, the emissive species could be produced, and the shoulder peak at 480 nm became prominent (Fig. 2b). After 10 min of photoirradiation, the powder XRD spectra of **NPBr** crystals revealed no discernible changes compared to their pristine state (Fig. S4), indicating that the molecular packing within the crystalline lattice remained unchanged. As illustrated in Fig. S5, this photoinduced luminescence exhibits irreversibility, as it persists after heating or cessation of irradiation.

### Photoinduced luminescence mechanism

**Fig. 3:**
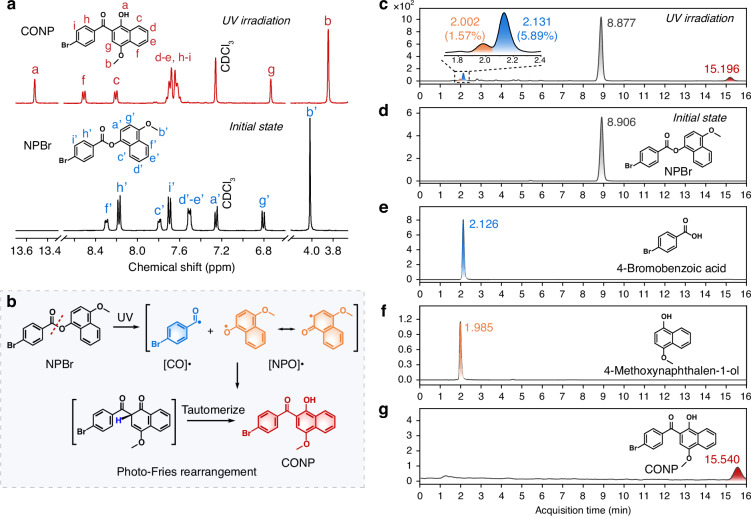
The mechanism of photoinduced luminescence. **a** ¹H NMR spectra of the pristine **NPBr** (bottom) and the isolated product **CONP** in CDCl_3_ (top). **b** The reaction mechanism for the photo-Fries rearrangement of **NPBr** in the solution phase upon UV irradiation. HPLC analysis of **d** pristine **NPBr** and **c** its photoirradiated products alongside **e** 4-bromobenzoic acid, **f** 4-methoxynaphthalen-1-ol, and **g** isolated product CONP using a hexane/chloroform (2:3) mobile phase on a Daicel Chiralpak IE column

UV irradiation is generally known to disrupt molecular structures^35–37^; hence, assessing the structural integrity of the molecules is essential. Figure S6 illustrates the emergence of new ^1^H NMR signals at δ 12.67, 8.44, 6.81, and 3.85 ppm after 30 min of UV exposure, indicating that the **NPBr** molecules underwent a photochemical reaction. To elucidate the photochemical reaction pathways, the UV-irradiated **NPBr** solution was separated and purified by preparative thin-layer chromatography (PTLC), yielding a small amount of isolable product. Its single crystals were subsequently obtained via slow solvent evaporation. The ¹H NMR spectrum of this product (Figs. 3a and S7) displays notable changes in the chemical shifts of aromatic protons compared to those of **NPBr**. In addition to the original Hb’–Hi’ signals, a new proton resonance, denoted Ha, is observed. Combined with single-crystal XRD analysis (Fig. S8), the formation of **CONP** after UV irradiation was confirmed. This structural identification provides clear evidence that UV irradiation induces homolytic cleavage of the ester bond in **NPBr**, generating [CO]• and [NPO]• radicals. These radical species subsequently undergo a photo-Fries rearrangement in solution, ultimately leading to the formation of rearrangement product **CONP** (Fig. 3b). To further characterize the radical intermediates and quantify their abundance, high-performance liquid chromatography (HPLC) was performed using a Poroshell column. While pristine **NPBr** showed a retention time of 8.906 min (Fig. 3d), the irradiated sample showed three new peaks at 2.002, 2.131, and 15.196 min (Fig. 3c). These retention times are analogous to those of 4-bromobenzoic acid, 4-methoxy-naphthalen-1-ol, and **CONP**, respectively (Fig. 3e–g), thus confirming the identity of the radical-derived fragments and corroborating the reaction mechanism inferred from single-crystal XRD analysis. It is noteworthy that the infrared (IR) spectra of **NPBr** crystals revealed negligible change after UV irradiation for 1 h (Fig. S9), suggesting that photodissociation occurred only in a small fraction of these molecules. This proposal is consistent with quantitative HPLC results. The photophysical properties of **NPBr** and its photoreaction products were subsequently compared. As shown in Figs. S10a and S11a, 4-bromobenzoic acid and 4-methoxynaphthalen-1-ol displayed the absorption peaks at 270 and 284 nm, respectively, which differ significantly from the absorption peak at 441 nm observed for UV-irradiated **NPBr** powder. Moreover, 4-bromobenzoic acid emitted phosphorescence with PL peaks at 510 and 548 nm (Fig. S10b–d), whereas 4-methoxynaphthalen-1-ol and **CONP** showed PL peaks at 386 and 583 nm, respectively (Figs. S11b and S12). These results indicate that the broad shoulder peak at 480 nm in the PL spectrum of irradiated **NPBr** cannot be attributed to 4-bromobenzoic acid, 4-methoxynaphthalen-1-ol, or CONP. Therefore, the luminescence of photoirradiated **NPBr** powder is unlikely to originate from protonation or rearrangement products of the radicals. Instead, it is reasonable to attribute the observed photoinduced luminescence to emission from [CO]• or [NPO]• radicals generated under UV irradiation.

To obtain direct evidence for the presence of these proposed radical species, we performed EPR spectroscopic studies. As depicted in Fig. 2f, a weak but persistent EPR signal at *g* = 2.0040 was observed in UV-irradiated **NPBr** crystals, suggestive of radical formation. The persistence of this signal, even after removal of the irradiation (Fig. S13), aligns with the observed irreversible fluorescence switching, suggesting the generation of stable radical species. To further confirm the identity of the radical species, EPR spin-trapping experiments were conducted using 5,5-dimethyl-1-pyrroline N-oxide (DMPO). No EPR signals were detected prior to irradiation, but a multiline spectrum emerged after irradiation at 300 K (Fig. S14a, b). The observed signals were assigned to DMPO adducts of the benzoyl radical ([CO]•, with hyperfine coupling constants *A*_N_ = 14.06 G and *A*_H_ = 15.81 G) and the hydroperoxyl radical ([NPO]•, with *A*_N_ = 12.86 G and *A*_H_ = 10.01 G), consistent with reported values for DMPO-trapped species. The experimental spectrum was successfully simulated as a superposition of DMPO/[CO]• and DMPO/[NPO]• adducts (Fig. S14c–e). When the naphthalene moiety of **NPBr** was replaced by a phenyl group, the resulting compound, benzoate ester **BzO**, exhibited a broad, less intense emission at 472 nm after UV irradiation (Fig. S15). This PL intensity was significantly lower than that observed for photoirradiated **NPBr**. Moreover, simulation studies further suggested that the radical signal detected in the DMPO/BzO system in CH_2_Cl_2_ solution was indeed a combination of DMPO/[CO]• and DMPO/[BPO]• adducts (Fig. S16). We also synthesized two control molecules, **NA** and **BrO**, by replacing the bromine atom and the methoxy group in **NPBr** with hydrogen atoms, respectively. The crystalline powder of **NA** exhibited photoinduced luminescence properties and fluorescence lifetime similar to **NPBr**, while **BrO** showed no fluorescence at all (Fig. S17). These findings showed that the emerging PL peak at 480 nm observed in photoirradiated **NPBr** correlated with the [NPO]• radical. The TD-DFT calculations were performed using the M06-2X (6–31 G(d,p)) functional to further investigate the radical emission. The optimized energy gap for the *D*_0_ → *D*_*n*_ (*n* = 2 and 3) transition of the oxygen-centered radical ([NPO]•) has been calculated to be 2.69 eV (461 nm) and 2.78 eV (446 nm), with corresponding oscillator strengths (*f*) of 0.0220 and 0.0560. These values are remarkably close to the absorption peak observed at 441 nm in photoirradiated **NPBr** powders (Fig. 2a). However, the benzoyl radical ([CO]•) exhibited the smallest energy gap of 3.3 eV for *D*_0_ → *D*_n_ (*n* = 2, 3, and 4) electronic states, and the corresponding transitions were nearly forbidden (*f* < 0.0003). The results further confirm that the oxygen-centered radical ([NPO]•) is responsible for the photoinduced radical emission. However, the photogenerated radicals are unstable in CH_2_Cl_2_ due to the absence of spatial confinement, which is present in both crystalline and rigid PMMA matrices. Experimental evidence supports this conclusion: When **NPBr** was dissolved in CH_2_Cl_2_ at a low concentration (1 mg mL^−1^), no EPR signal was detected upon photoirradiation at room temperature (Fig. S18a), but it could be detected at 120 K (Fig. S18b). This indicates that the photogenerated radicals are unstable in dilute solution and can only be detected when their decay and recombination processes are kinetically inhibited. Notably, an observable room-temperature EPR signal required increasing the **NPBr** concentration to 68 mg mL^−1^ (Fig. S18c). Moreover, the PL spectrum for this concentrated solution exhibits a peak at 517 nm upon photoirradiation, and the fluorescence intensity decreases gradually over time after the photoirradiation is discontinued (Fig. S18d). This time-dependent fluorescence quenching, paralleled by a concurrent decay of the EPR signal (Fig. S18c), consistently indicates the instability and transient nature of the radicals in the solution phase.

Based on comprehensive experimental evidence, we propose a photoactivated self-doping mechanism to account for the photoinduced radical luminescence in benzoate ester. Upon photoirradiation, a small fraction of **NPBr** molecules undergo a photodissociation process, generating limited benzoyl ([CO]•) and hydroperoxyl ([NPO]•) radicals (approximately 5% molar ratio by HPLC). Photophysical testing and theoretical calculations indicate that the [NPO]• radicals exhibit intense blue fluorescence (*λ*_em_ = 480 nm, PLQY = 47.7%), which dominates the observed photoinduced radical luminescence (Fig. 2b). Crucially, compared to a liquid-phase environment, the **NPBr** crystalline matrix effectively isolates individual radical species through spatial confinement. This isolation mechanism suppresses radical recombination pathways, enabling remarkable stability of the emissive species over 30 days under ambient conditions (Fig. S5). The irreversible character of the photoinduced luminescence aligns with a self-doping mechanism: (i) Localized photolysis creates isolated [NPO]• radicals embedded within the host **NPBr** lattice; (ii) These emissive dopants remain spatially fixed due to the restricted molecular mobility in the crystalline state (XRD analysis, Fig. S4), effectively preventing quenching and rearrangement. This photoactivated self-doping process differs fundamentally from conventional physical doping strategies, as it easily achieves spatial control of dopant distribution through the photodissociation process.

### Flexibility of crystals

**Fig. 4:**
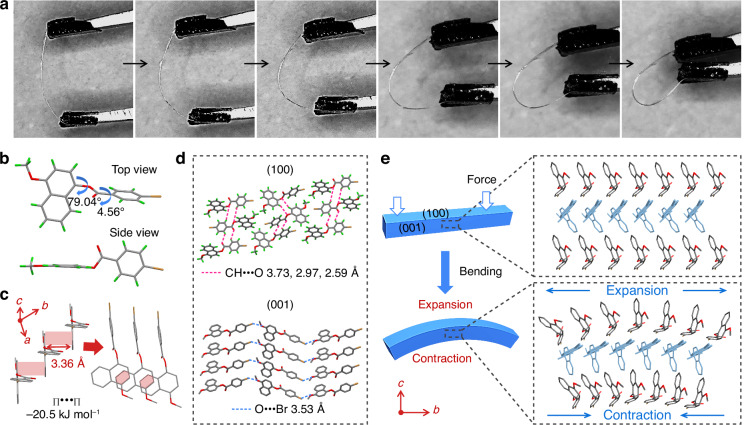
Flexibility and crystal packing features of NPBr crystals. **a** Elastic bending process of a needle-shaped **NPBr** crystal compressed by tweezers. **b** Molecular conformation of the **NPBr** crystal. **c** The overlapping area and vertical distance of the π−π interaction along one-dimensional molecular chain. **d** Crystal packing diagrams viewed along the *a*- and *c*-axes directions, revealing the hydrogen-bonded network that constructs the (100) and (001) crystallographic planes. **e** Schematic representation of crystal deformation mechanisms: (top) pristine single crystal and (bottom) bent morphology. The arrows indicate the expansion and compression directions of outer and inner arcs during the bending process

Long crystalline needles were readily obtained, and their photophysical properties were investigated. The typical lengths of the crystals were 10–20 mm with widths ranging from 0.4 to 1 mm. Similar to the crystalline powders, the needle-shaped **NPBr** crystals exhibited photoinduced luminescence. Notably, these crystals also displayed remarkable elasticity. As depicted in Fig. 4a, applying stress to the ends of a straight crystal using tweezers induced bending along the widest faces, exceeding 180° without cracking or breaking. Upon the release of the applied stress, the crystal instantaneously reverted to its original, straight configuration (Fig. S19 and Video S1). This bending-relaxation cycle could be repeated several times without apparent degradation, demonstrating the remarkable elastic resilience of **NPBr** crystals. In addition, the corresponding luminescent behavior remained unchanged upon mechanical deformation. To quantitatively assess the mechanical properties of the flexible **NPBr** crystals, nanoindentation measurements were conducted (Fig. S20). The average Young’s modulus of **NPBr** crystals was determined to be approximately 9.76 GPa, which was very close to that of the crystals after photoirradiation (9.50 GPa). This suggests that photoirradiation does not significantly alter the mechanical properties of the crystals. Consistent with these findings, UV-irradiated **NPBr** crystals exhibited good flexibility and could be readily bent under external force, as depicted in Fig. S21.

To elucidate the flexible nature of **NPBr**, its crystal structure was determined using single-crystal XRD analysis. The crystal belongs to the monoclinic space group *P*2_1_/*n* (*Z* = 4, *ρ* = 1.635 cm³). The asymmetric unit comprised one **NPBr** molecule, with no solvent molecules present within the crystal lattice. Each **NPBr** molecule exhibited a bent conformation, evidenced by a dihedral angle of approximately 90° between the phenyl ring and the naphthalene unit (Fig. 4b). And the neighboring molecules revealed a distinct overlap between naphthalene planes with a short distance of approximately 3.36 Å (Fig. 4c). The neighboring π-π stacking had significant interaction energy (−20.5 kJ mol^−1^), driving molecular chain formation along the *b*-axis. As depicted in Figs. 4d and S22, these molecules were connected along the *c*-axis direction by two types of CH•••O interactions with distances of 2.59 and 3.73 Å, which produce a structurally stable molecular layer. Moreover, these stacked columns were linked by intermolecular hydrogen bonds CH•••O and Br•••O halogen bond with the distance of 2.97 Å and 3.53 Å along the [010] direction, generating the bendable (001) planes. Consequently, in addition to the infinite π-stacking, the C–H•••O hydrogen bonds (−17.7 kJ mol^−1^) and Br•••O halogen interactions (−5.1 kJ mol^−1^) between molecular chains suppressed the breaking tendency of molecular chains towards expansion, thus benefiting the high elasticity of OSCs.

To verify the critical role of Br•••O interactions, we performed crystal structure analysis on **NA** and **BrO**. Both **NA** and **BrO** crystallized as block-shaped crystals, sharply contrasting with the needle-like morphology of **NPBr** (Fig. S24). Single-crystal analyses revealed the absence of Br•••O interactions in these derivatives, accompanied by a complete disruption of the directional molecular chains observed in **NPBr**. The packing arrangements of **NA** and **BrO** exhibited disordered stacking patterns with increased intermolecular distances (Fig. S25), confirming that halogen bonding is essential for maintaining the one-dimensional (1D) chain architecture. These comparative results conclusively establish that Br•••O interactions serve as key structural determinants for both crystal morphology and elastic deformability in this system^6^. Why do **NPBr** crystals exhibit controllable elasticity and bending? As illustrated in Fig. 4e, the elastic bending of the crystal involved the respective expansion and contraction of the outer and inner arcs. As molecular chains aligned along the *b*-axis, the π-π interactions within these chains dynamically adjusted to the outer arc’s expansion by increasing the π–π distance, while responding to the inner arc’s contraction by decreasing the π–π distance. In the crystal structure of the (010) plane, the oxygen atoms form type-II O/Br contacts at a distance of 3.53 Å and exhibit C–H/O interactions at distances of 2.97 Å (Fig. S23). The multiple Br–O and C–H/O interactions, which are arranged in diverse directions and balanced with each other, are relatively stable and thus could avoid changing the position of the molecule, i.e., preventing displacement of the molecular layer structure, during the process of elastic bending. Consequently, these interactions play a critical role in enabling crystal elasticity^41–43^.

### Optical waveguiding properties

**Fig. 5:**
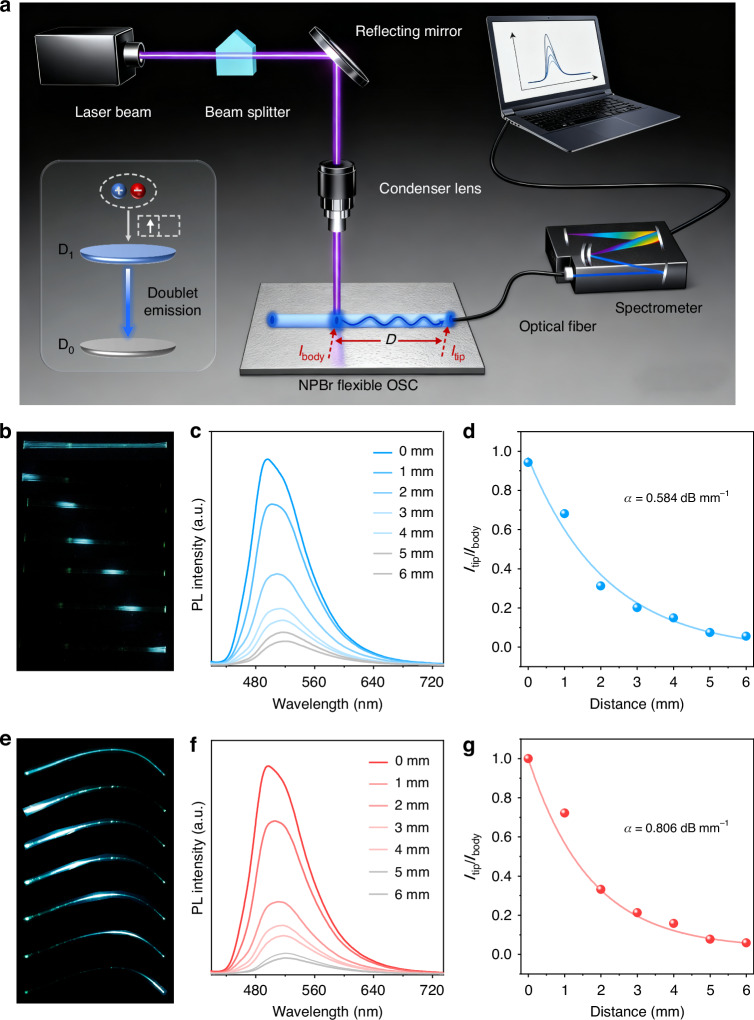
Optical waveguiding properties of NPBr crystals. **a** Schematic diagram of the optical waveguide test setup. **b**, **e** Straight and bent crystals under excitation from a UV lamp (top image) and a 355 nm laser focused on different positions. **c**, **f** PL spectra collected at the tip of a single crystal in the straight state and bent state with the different distances between the tip and the excitation site of the laser. **d**, **g** The *I*_tip_/*I*_body_ decay curves of a single crystal (length = 6 mm) in the straight and bent states. The optical loss coefficients (*α*) were determined using a single exponential fit to the equation *I*_tip_/*I*_body_ = *A*exp(−*αD*), where *I*_tip_ and *I*_body_ represent the PL intensities of out-coupled and incident light, respectively

Interestingly, the crystal exhibits optical waveguiding properties, which were observed from the brighter emission at the tips compared to the body when the straight crystal was illuminated with a UV lamp. Further investigations revealed that this waveguiding behavior is not limited to the straight crystal but is also present in highly bent crystals. To quantitatively evaluate the effects of elastic bending on waveguiding performance, the optical loss coefficients (OLCs) of a single **NPBr** crystal were investigated in both its straight and highly bent configurations, as illustrated in Fig. 5a. By exposing various positions along the crystal to a consistent excitation source of a 355 nm laser (Fig. 5b, e) and subsequently collecting the emission spectra at one tip of the crystal, distance-dependent PL profiles were established as depicted in Fig. 5c, f. The PL intensity at the tip diminished progressively as the distance between the tip and the irradiated position increased, attributed to a greater loss of emitted light over longer propagation distances. Additionally, the PL spectra exhibited a slight redshift with increased propagation distance, caused by enhanced reabsorption within the spectral overlap region between PL and absorption bands (Fig. S26). Utilizing fitting procedures described in the literature for the data presented in Fig. 5d, g^44^, the OLCs at 488 nm were estimated to be 0.584 dB mm^−1^ for the straight state and 0.806 dB mm^−1^ for the bent state. These low OLC values surpass those of many reported organic crystals and polymeric systems^45–49^, indicating a favorable waveguiding performance in both configurations. Notably, the OLC for the bent state is slightly increased relative to that of the straight state. The maintenance of a low OLC in the highly bent condition is particularly intriguing, as optical losses in waveguides are typically exacerbated in bent configurations.

## Discussion

In summary, we have demonstrated a novel photoinduced radical self-doping strategy to integrate radical luminescence and mechanical flexibility in OSCs. The naphthyl benzoate derivative **NPBr** forms needle-like crystals that undergo UV-triggered photodissociation, generating stable oxygen-centered radicals ([NPO]•) with intense blue luminescence. Crucially, the crystalline matrix confines radicals at low concentrations (approximately 5 mol%), suppressing recombination while preserving structural integrity. Impressively, this photoinduced radical doping process enables simultaneous radical luminescence and elastic deformability, sustained by dynamic π–π stacking (−20.5 kJ mol^−1^) and Br•••O halogen bonding. Single-crystal analyses reveal that directional 1D molecular chains and noncovalent interactions synergistically dissipate mechanical stress, yielding a Young’s modulus of ~9.76 GPa. Control experiments with non-halogenated analogs confirm the indispensability of Br•••O interactions for chain formation and flexibility. Additionally, a low-loss optical waveguide was achieved in both straight and bent crystals, which shows a potential optical application. This work establishes a paradigm for the design of flexible OSCs with radical luminescence, offering a versatile platform for next-generation optoelectronic and photonic devices.

## Materials and methods

### Characterization

^1^H and ^13^C NMR spectra were obtained using Brüker AM400 (400 MHz for ^1^H NMR and 101 MHz for ^13^C NMR) spectrometer with tetramethylsilane (TMS, *δ* = 0 ppm) as internal reference. Chemical shifts are reported in δ ppm using DMSO-*d*_6_ (2.54 ppm) for ^1^H NMR and DMSO-*d*_6_ (39.53 ppm) for ^13^C NMR as an internal standard. The following abbreviations were used to explain the multiplicities: *s* = singlet, *d* = doublet, *t* = triplet, *m* = multiplet. High-resolution mass spectrometry (HRMS) analysis was carried out on a Thermo Scientific Exactive LC-MS system with an atmospheric-pressure chemical ionization source. The HPLC analysis was conducted using an Agilent 1260 LC system coupled with an Agilent G6230 Time-of-Flight mass spectrometer. The UV-irradiated **NPBr** sample refers to exposure to 365 nm UV light (38.5 mW cm^−2^) for 10 min. Single-crystal XRD data collection was performed on a Bruker SMART APEX II CCD diffractometer with CuKα radiation (*λ* = 1.54178 Å, graphite monochromator). UV-vis absorption profiles were obtained from a Shimadzu UV-2600 spectrophotometer using quartz cuvettes (1 cm path length). FT-IR spectra of **NPBr** were acquired on a Nicolet NEXUS670. The photoirradiation was performed using a TK566 (D1) UV lamp (3 W nominal power) emitting monochromatic light at 365 nm (LED-based source, no secondary emission maxima). The lamp-to-sample distance was maintained at 10–15 cm, and the power density at the sample position was measured as 38.5 mW cm^−2^ using a calibrated optical power meter. This setup ensured spectral purity (single wavelength at 365 nm) and uniform illumination. All optical images were documented using a Canon 550D digital camera with standardized illumination conditions.

### EPR

Quantitative EPR measurements were acquired at room temperature on a Bruker EMX-nano spectrometer (Bruker BioSpin). 5,5-dimethyl-1-pyrrolidine N-oxide (DMPO) was selected as the trapping agent in the EPR test. A reference-free quantitative EPR function was implemented in the Xenon® software package on EMX-nano. The absolute spin value was calculated using pre-defined microwave bridge calibration, resonator profile, and measured *Q* value and signal integration. The EPR settings were as follows: microwave power, 20 mW; total acquisition time, 7 min; magnetic field modulation, 1 G; sweep width, 150 G; centerfield, 3520 G; sweep time, 60 s; sweep width, 150 G.

### Photophysical properties

PL spectra and time-resolved PL decay curves were performed with an Edinburgh FLS 980 fluorescence spectrophotometer equipped with three excitation sources: (i) 450 W xenon arc lamp (Xe900) for steady-state measurements, (ii) nanosecond hydrogen flashlamp (*n*F920, 1–20 ns pulse width), and (iii) microsecond flashlamp (*μ*F900, 10–100 μs pulse width) for time-resolved PL studies of NPBr. Absolute PLQYs were quantified using a Hamamatsu C11347 integrating sphere spectrometer under a nitrogen atmosphere.

### Mechanical properties

Mechanical properties were evaluated via nanoindentation using a Bruker Hysitron TI Premier system equipped with a Berkovich diamond tip (100 nm radius), applying a 2 mN s^−1^ loading rate.

### Optical waveguiding tests

A crystal was placed on a silicon wafer, with one end of the crystal extending beyond the edge of the wafer and aligned with the probe of the fiber-optic spectrometer. The sample was locally excited by the third harmonic (355 nm) of a Nd:YAG laser, which was focused into a small spot of ~500 μm through a convex lens and a slit. By moving this focused excitation laser beam to different positions along the crystal body, the emission spectra were collected locally at the excitation point and the crystal tip using a Maya2000 Pro CCD spectrometer. After the collection, the crystal was bent and fixed with glue, and the emission spectra were collected using the same method mentioned above.

## Supplementary information

Supplementary Materials

Video S1-Elastic bending process of NPBr crystal

CIF of NPBr crystal

CIF of NA crystal

CIF of BrO crystal

CIF of CONP crystal

## Data Availability

The data that support the findings detailed in this study are available in the Supplementary Information or from the corresponding authors upon reasonable request.
