# Supplementary material for: Photoinduced radical emission from flexible organic crystals
Source: Light Sci Appl. 2026 May 19;15:240. doi: 10.1038/s41377-026-02208-6 (PMC13187443; doi:10.1038/s41377-026-02208-6)
Supplement: Supplementary file 1 — Supplementary Materials [file 41377_2026_2208_MOESM1_ESM.pdf]

Supplementary Information

Photoinduced radical emission from flexible organic crystals

Xuan Zhang[1,†], Wenyuan Pan[1,†], Yuqi Tang[2,†], Ting Huang[1], Xuesong Yang[3], Hui Mao[1], Feiying Ruan[1], Qing Luo[1], Lin Li[1], Hongyu Zhang[3,*], Yujian Zhang[1,*] and Quan Li[2,4,*]

[1]Key Laboratory of the Ministry of Education for Advanced Catalysis Materials, Department of Chemistry, Zhejiang Normal University, Yingbin Road NO.688, Jinhua 321004, China

[2]Institute of Advanced Materials, School of Chemistry and Chemical Engineering, and School of Electronic Science & Engineering, Southeast University, Nanjing 211189, China

[3]State Key Laboratory of Supramolecular Structure and Materials, College of Chemistry, Jilin University, Qianjin Street NO. 2699, Changchun 130012, China

[4]Materials Science Graduate Program, Kent State University, Kent, OH 44242, USA

†These authors contributed equally to this work

*Correspondence: Yujian Zhang (sciencezyj@foxmail.com); Hongyu Zhang (hongyuzhang@jlu.edu.cn); Quan Li (quanli3273@gmail.com)

1. Preparation of chromophores

1.1 Synthesis of NPBr

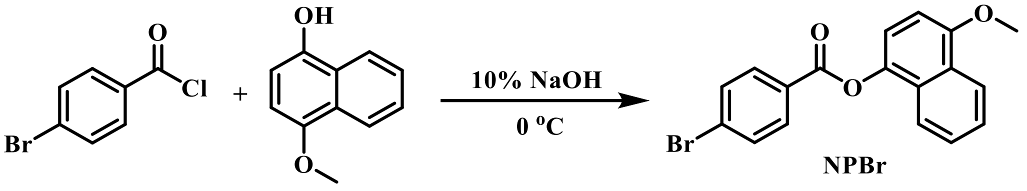

Scheme S1. The synthetic route of **NPBr**.

In a 50 mL round-bottom flask, 4-methoxy-1-naphthol (0.87 g, 5 mmol) was dissolved in 20 mL of 10% sodium hydroxide solution. The mixture was stirred in an ice bath for 15 min. Then 4-bromobenzoyl chloride (1.32 g, 6 mmol) dissolved in acetonitrile was added and the reaction proceeded for 2 h. The crude product was purified by column chromatography on silica gel and eluted with petroleum ether and dichloromethane (volume ratio = 3:1) to obtain white solids (1.62 g) with a yield of 76%. **^{1}H NMR** (400 MHz, DMSO-d_6) δ 8.25-8.20 (m, 1H), 8.19-8.15 (m, 2H), 7.89-7.85 (m, 2H), 7.80-7.75 (m, 1H), 7.61-7.55 (m, 2H), 7.40 (d, J = 8 Hz, 1H), 7.02 (d, J = 8 Hz, 1H), 4.01 (s, 3H); **^{13}C NMR** (101 MHz, DMSO-d_6) δ 164.92, 153.45, 139.88, 132.73, 132.34, 128.79, 128.47, 127.84, 126.52, 125.82, 122.46, 121.32, 119.02, 104.17, 56.37; **HRMS** (ESI, m/z) Calculated for $C_{18}H_{13}O_3Br$ = 357.0121, found $[M]^+$ = 357.0112.

1.2 Synthesis of NA

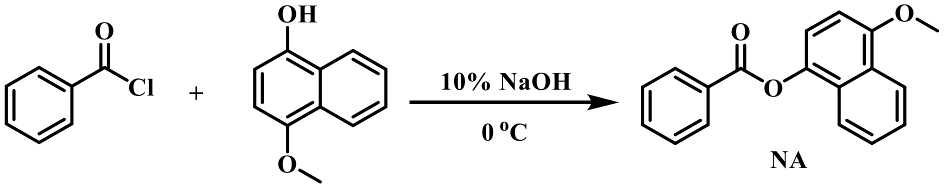

Scheme S2. The synthetic route of **NA**.

In a 50 mL round-bottom flask, 4-methoxy-1-naphthol (1.1 g, 2.5 mmol) was dissolved in 20 mL of 10% sodium hydroxide solution. The mixture was stirred in an ice bath for 15 min. Then benzoyl chloride (1.68 g, 3 mmol) dissolved in acetonitrile was added and the reaction proceeded for 2 h. The crude product was purified by column chromatography on silica gel. Elution with petroleum ether and dichloromethane (3:1) afforded white solids (0.38 g) in a yield of 54%. **^{1}H NMR** (400 MHz, DMSO-d_6) δ 8.30-8.21

(m, 3H), 7.83-7.76 (m, 2H), 7.66 (m, J = 10.7, 4.7 Hz, 2H), 7.61-7.56 (m, 2H), 7.40 (d, J = 8 Hz, 1H), 7.02 (d, J = 8 Hz, 1H), 4.02 (s, 3H). **^{13}C NMR** (101 MHz, DMSO-d_6) δ 165.54, 153.39, 140.03, 134.65, 130.37, 129.59, 129.24, 127.80, 127.50, 126.48, 125.84, 122.49, 121.31, 119.03, 104.19, 56.35. **HRMS** (ESI, m/z) Calculated for $C_{18}H_{14}O_3$ = 279.1016, found $[M]^+$ = 279.1014.

1.3 Synthesis of BrO

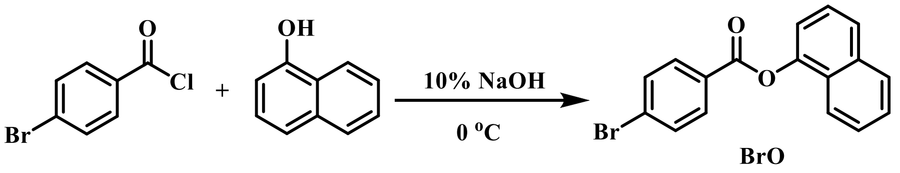

Scheme S3. The synethetic route of **BrO**.

In a 50 mL round-bottom flask, 1-naphthol (0.43 g, 3 mmol) was dissolved in 20 mL of 10% sodium hydroxide solution. The mixture was stirred in an ice bath for 15 min. Then, 4-bromobenzoyl chloride (0.79 g, 3.6 mmol) dissolved in acetonitrile was added and the reaction proceeded for 2 h. The crude product was purified by column chromatography on silica gel. Elution with petroleum ether and dichloromethane (3:1) afforded white solids (0.50 g) in a yield of 50%. **^{1}H NMR** (400 MHz, DMSO-d_6) δ 8.21-8.15 (m, 2H), 8.07-8.00 (m, 1H), 7.94-7.82 (m, 4H), 7.64-7.53 (m, 3H), 7.52-7.46 (m, 1H), 3.34 (s, 3H). **^{13}C NMR** (101 MHz, DMSO-d_6) δ 164.61, 146.70, 134.67, 132.76, 132.38, 128.91, 128.56, 128.35, 127.41, 127.20, 126.76, 126.69, 126.24, 121.33, 119.11. **HRMS** (ESI, m/z) Calculated for $C_{17}H_{11}BrO_3$ = 327.0015, found $[M]^+$ = 327.0013.

1.4 Synthesis of BzO

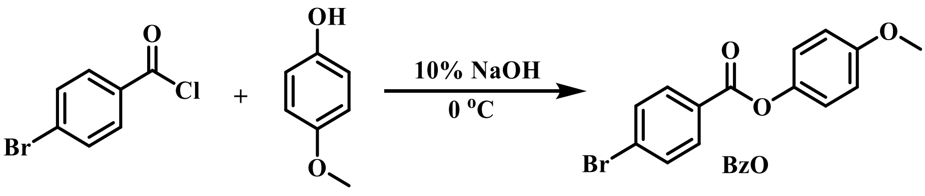

Scheme S4. The synethetic route of **BzO**.

In a 50 mL round-bottom flask, 4-methoxyphenol (0.62 g, 5 mmol) was dissolved in 20 mL of 10% sodium hydroxide solution. The mixture was stirred in an ice bath for 20 min. Then 4-bromobenzoyl chloride (1.32 g, 6 mmol) dissolved in acetonitrile was added and the reaction proceeded for 2 h. The crude product was purified by column

chromatography on silica gel. Elution with petroleum ether and dichloromethane (2:1) afforded white solids (0.54 g) in a yield of 35%. **^{1}H NMR** (400 MHz, $CDCl_3$) δ 8.07-8.02 (m, 2H), 7.67-7.61 (m, 2H), 7.14-7.09 (m, 2H), 6.97-6.90 (m, 2H), 3.81 (s, 3H). **^{13}C NMR** (101 MHz, $CDCl_3$) δ 164.95, 157.54, 144.33, 132.50, 132.03, 131.74, 128.84, 128.68, 122.46, 114.67, 55.72. **HRMS** (ESI, m/z) Calculated for $C_{14}H_{11}BrO_3$ = 306.9964, found $[M]^+$ = 306.9968.

2. Supplementary figures

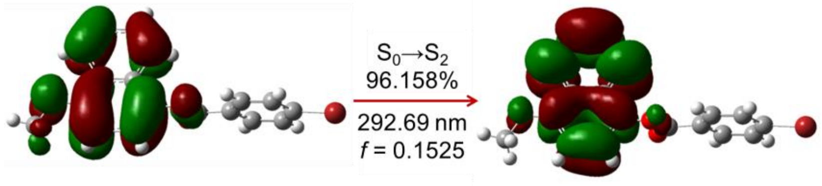

Figure S1. Natural transition orbital (NTOs) from S_0 to S_2 of **NPBr**, where f represents the oscillator strength.

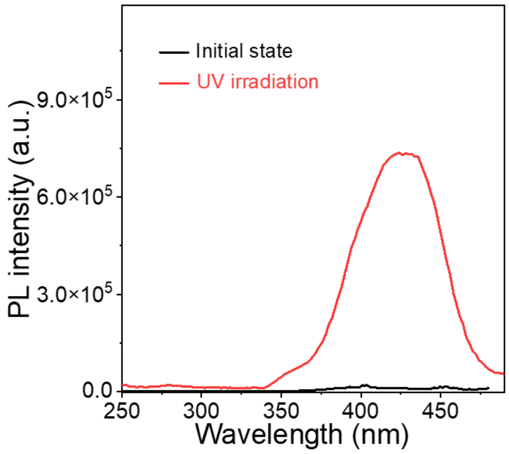

Figure S2. Excitation spectra of **NPBr** crystalline powders before (black curve) and after (red curve) 365 nm UV irradiation.

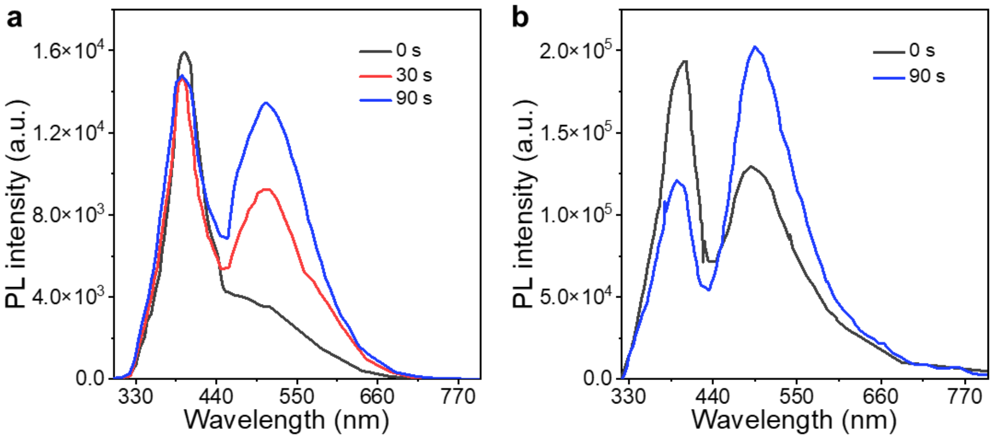

Figure S3. PL spectra of **NPBr** in (a) CH_2Cl_2 and (b) PMMA film as a function of irradiation time from 0 to 90 s.

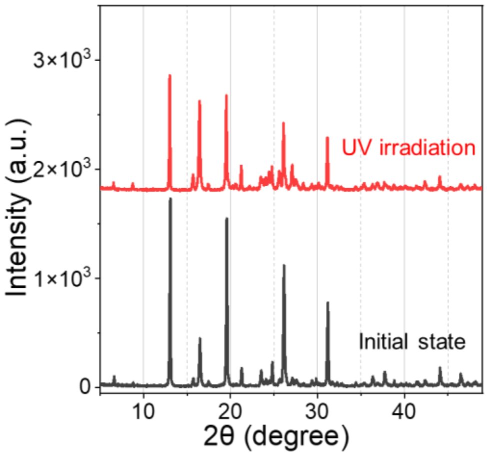

Figure S4. Powder XRD patterns of **NPBr** crystalline powders before (black curve) and after 365 nm UV irradiation (red curve).

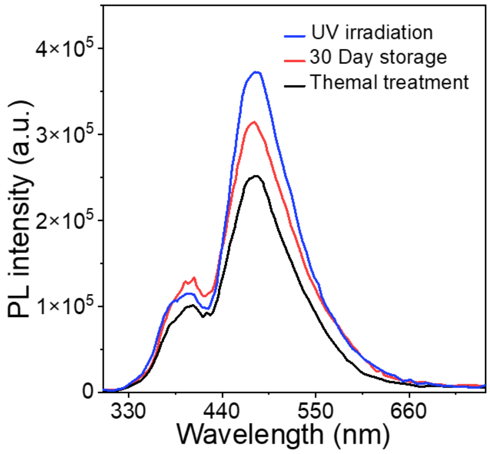

Figure S5. PL spectral evolution of **NPBr** crystals: pristine sample after 5-min UV irradiation (blue curve), the irradiated sample after 30-day ambient storage (red curve), and the photoirradiated sample upon 65 °C/10-min thermal treatment (black curve).

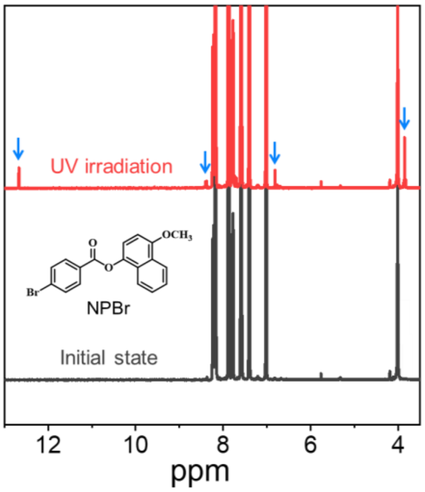

Figure S6. ^{1}H-NMR spectra of **NPBr** before and after 30 min UV irradiation.

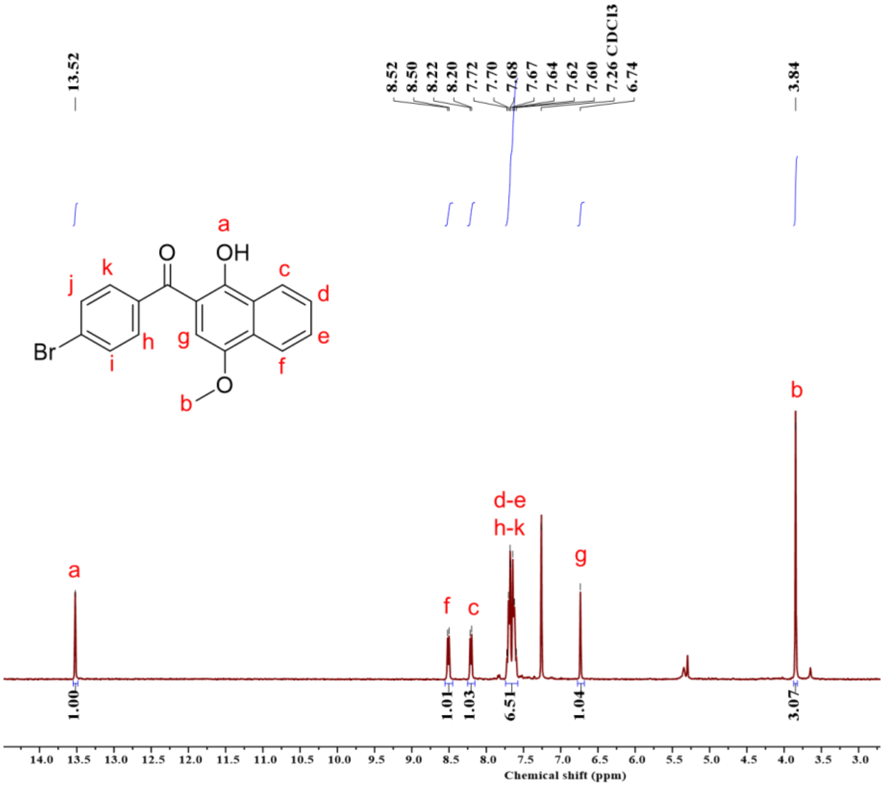

Figure S7. ^{1}H-NMR spectrum of the isolated product **CONP**.

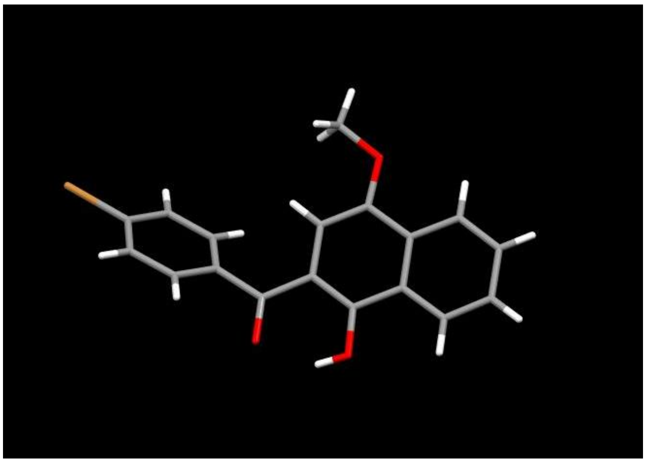

Figure S8. Single crystal structure of the isolated product **CONP**.

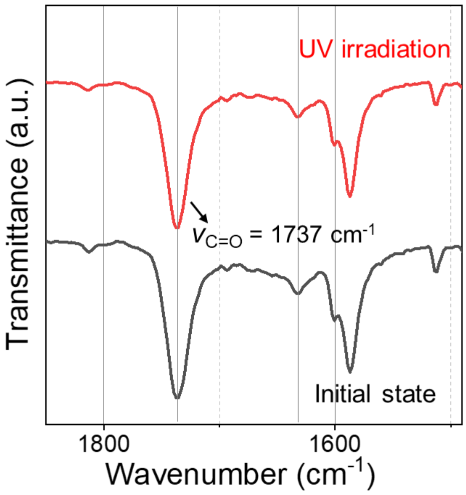

Figure S9. Fourier-transform infrared (FT-IR) spectral comparison between initial **NPBr** (gray trace) and UV irradiated samples (red trace).

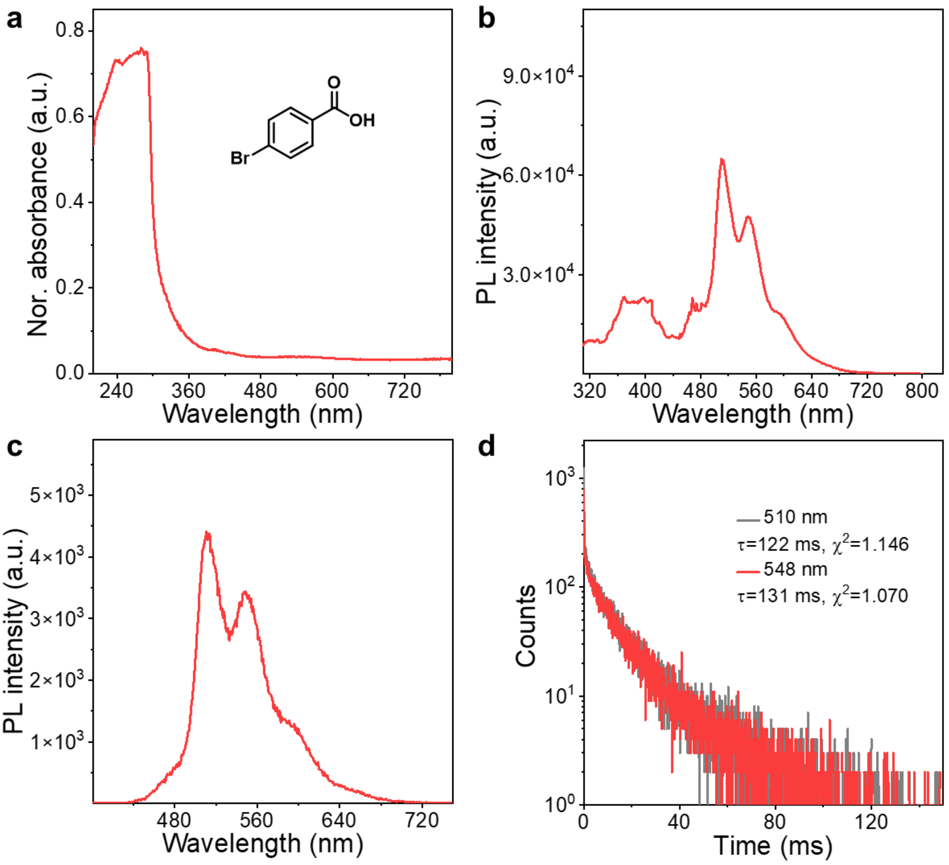

Figure S10. (a) Steady-state absorption spectrum, (b) PL spectrum and (c) delayed PL spectrum of 4-bromobenzoic acid crystalline powders. (d) Corresponding phosphorescence lifetime decay profile at 510 nm and 548 nm recorded under ambient conditions (25°C, air atmosphere).

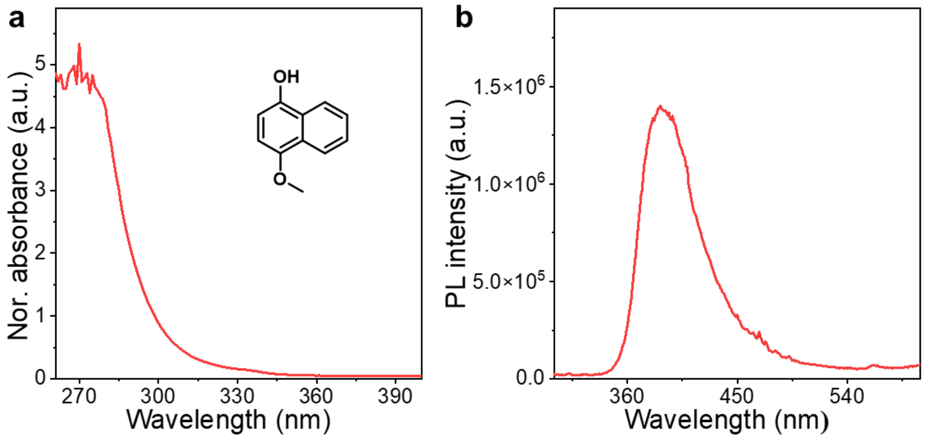

Figure S11. (a) Steady-state absorption spectrum and (b) PL spectrum of 4-methoxy-naphthalen-1-ol crystalline powders.

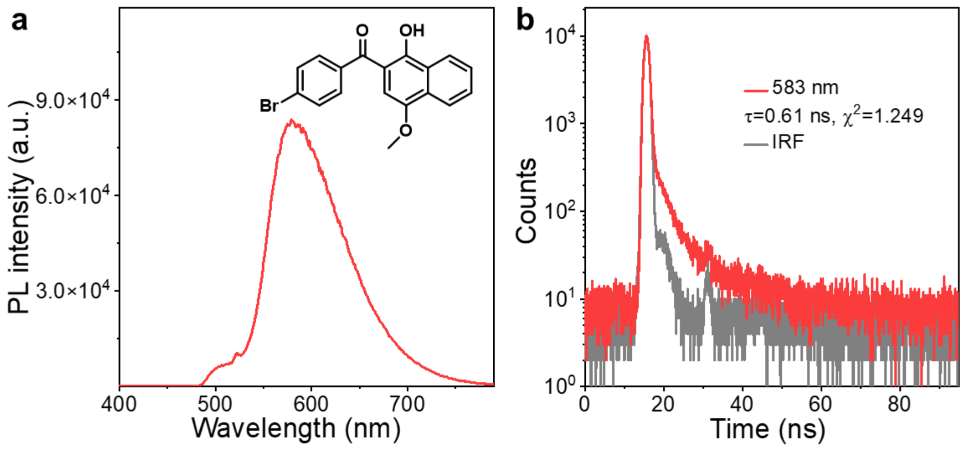

Figure S12. (a) PL spectrum and (b) time-resolved PL decay curves of **CONP** crystalline powders.

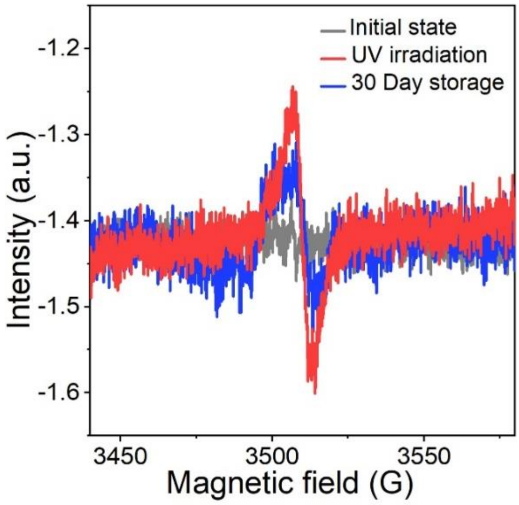

Figure S13. EPR spectral comparison between freshly photoirradiated **NPBr** (5-min exposure, red trace) and the irradiated sample after 30-day ambient storage (blue curve).

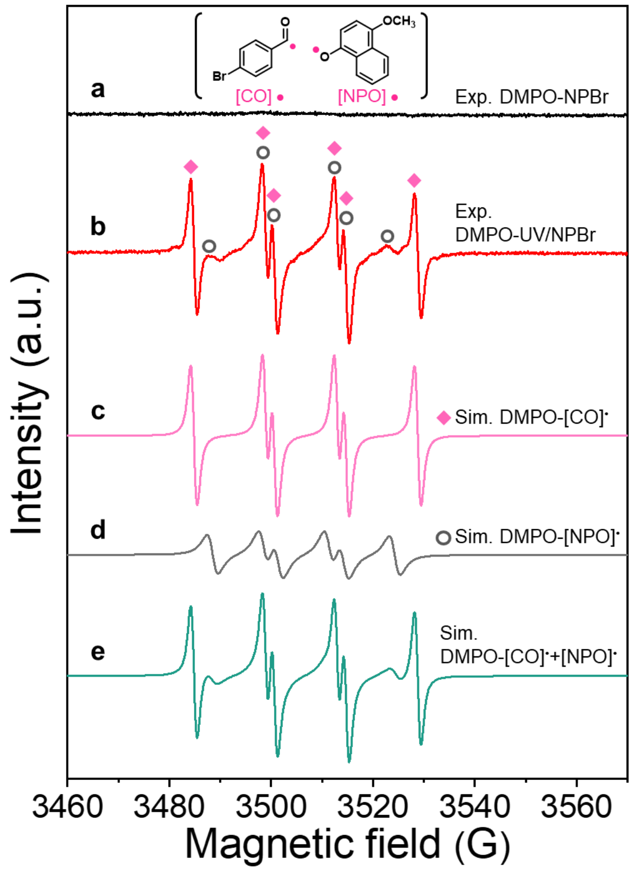

Figure S14. Experimental EPR spectra of **NPBr** in CH_2Cl_2 with DMPO (a) before and (b) after *in situ* photoirradiation (365 nm UV lamp, 38.5 mW cm^{-2}) during room-temperature detection. Simulated EPR spectra of (c) DMPO/[CO] • adduct, (d) DMPO/[NPO]• adduct, and (e) composite spectrum of DMPO adducts with [CO]• and [NPO]•.

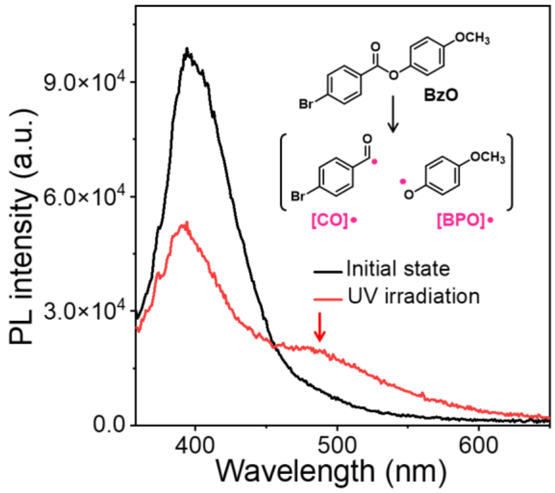

Figure S15. PL spectra of **BzO** before and after UV irradiation.

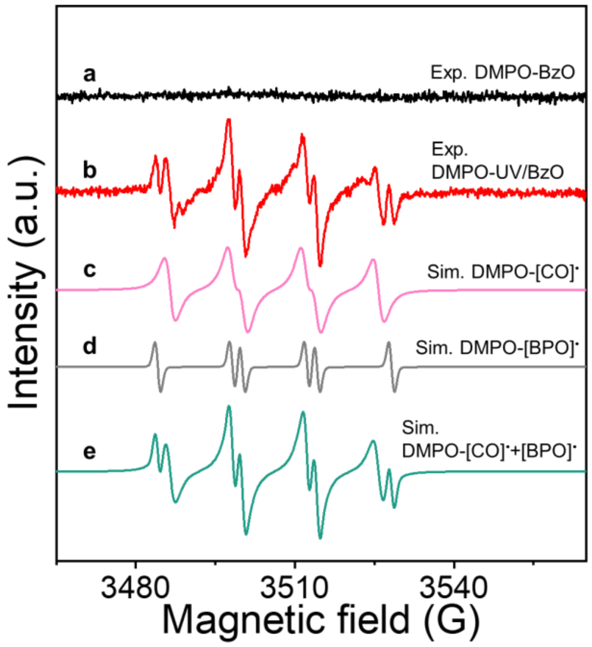

Figure S16. EPR spectra of **BzO** in CH_2Cl_2 with DMPO (a) before and (b) after *in situ* photoirradiation (365 nm UV lamp, 38.5 mW cm^{-2}) during room-temperature detection. Simulated ESR spectra of (c) DMPO/[CO]• adduct, (d) DMPO/[BPO]• adduct, and (e) composite spectrum of DMPO adducts with [CO]• and [BPO]•.

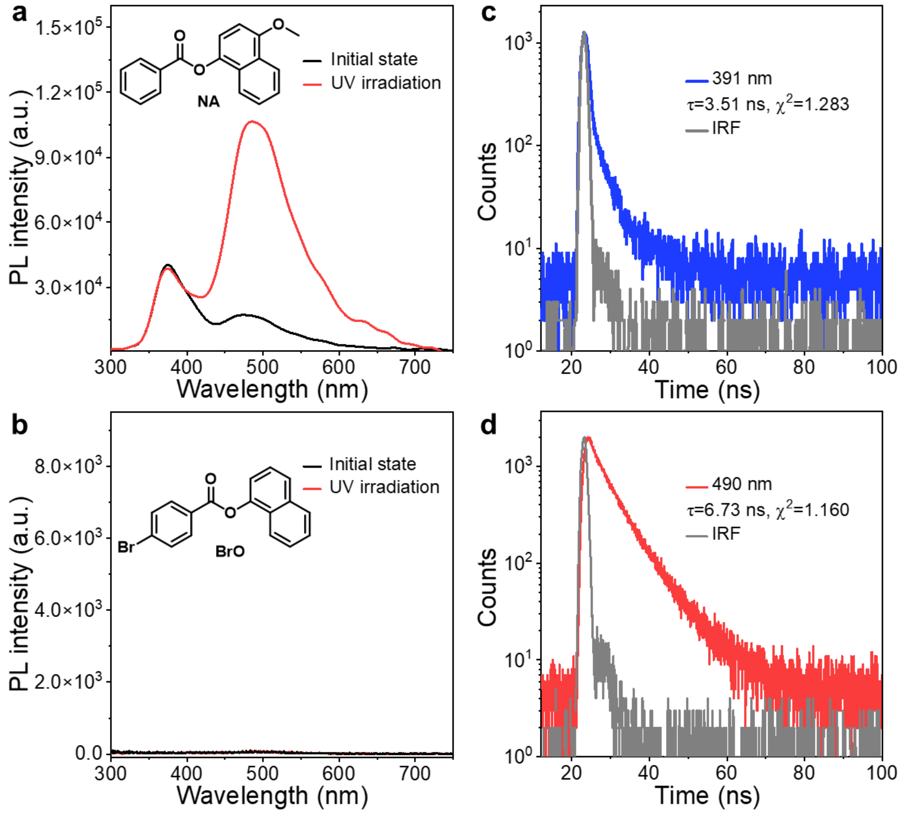

Figure S17. PL spectra of (a) **NA** and (b) **BrO** before and after UV irradiation. (c, d) Time-resolved PL decay curves of **NA** (λ_{em} = 391 nm and 490 nm) after UV irradiation.

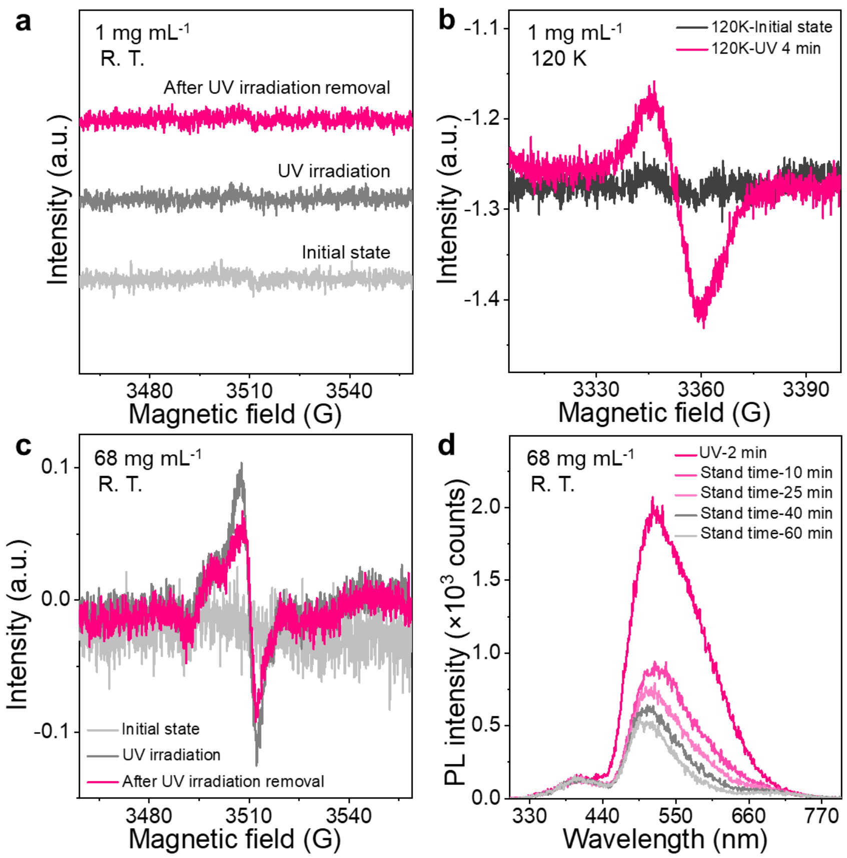

Figure S18. (a) Room temperature EPR spectra of **NPBr** dilute solution (1 mg mL^{-1}) in CH_2Cl_2. (b) Low-temperature EPR spectra of **NPBr** dilute solution (1 mg mL^{-1}) in CH_2Cl_2 recorded at 120 K. (c) EPR spectra of **NPBr** concentrated solution (68 mg mL^{-1}) in CH_2Cl_2 at room temperature. (d) Temporal evolution of PL spectra for **NPBr** in CH_2Cl_2 (68 mg mL^{-1}): Comparison between the pristine sample after UV irradiation for 2 min (deep-red curve) and the irradiated sample following dark storage for different durations (10-60 min).

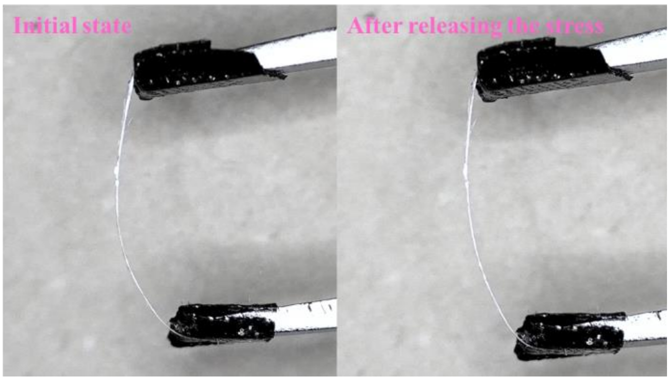

Figure S19. After the stress was released, the **NPBr** crystal returned to its original state without any changes.

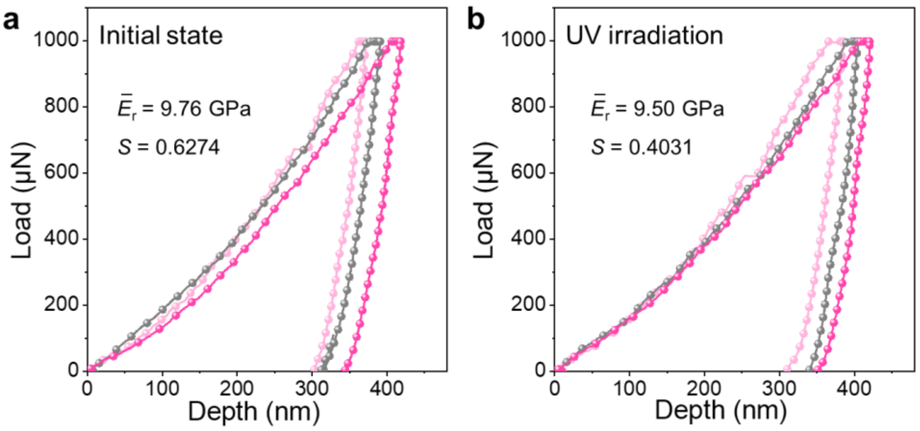

Figure S20. Nano-indentation test of **NPBr** crystal in (a) original state and (b) after UV irradiation. S (Standard deviations) = $\left(\frac{\sum_{i=1}^{n}(E_i-\bar{E_r})^2}{n-1}\right)^{1/2}$, E_i, $\overline{E_r}$ *and* n represents Young's modulus, average Young's modulus and the number of independent measurements. The Young's moduli of pristine **NPBr** crystals and UV-irradiated **NPBr** crystals were independently measured three times for each sample.

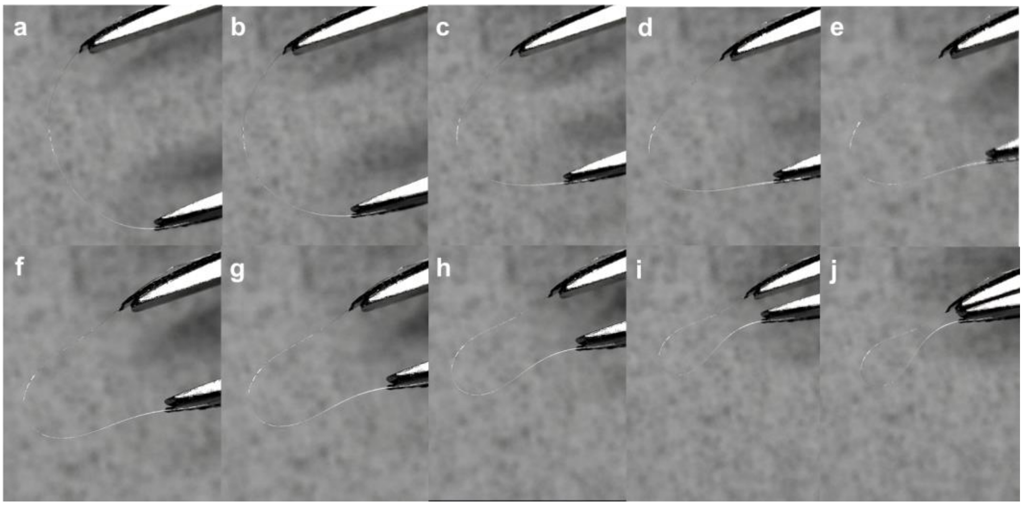

Figure S21. Elastic bending process of **NPBr** crystal compressed by tweezers.

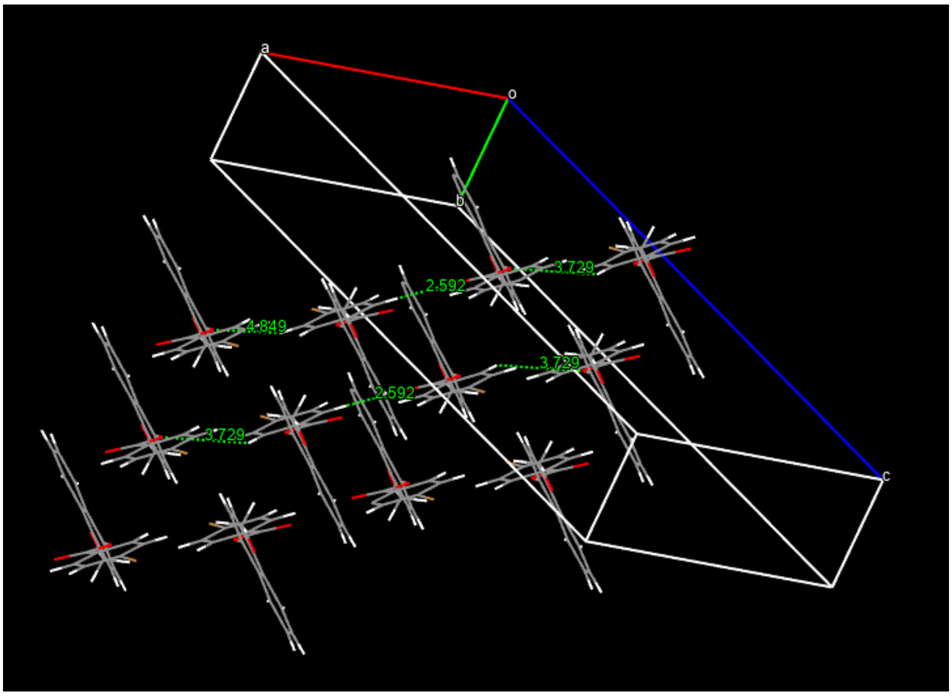

Figure S22. NPBr molecules were connected along the [101] direction by two types of CH•••O interactions with distances of 2.592 and 3.729 Å.

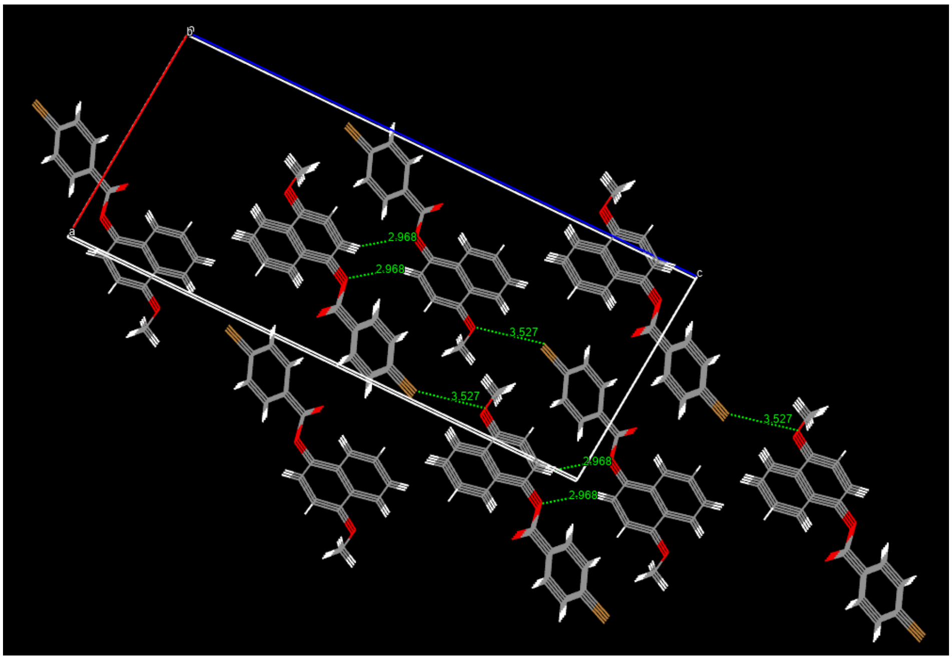

Figure S23. NPBr stacked columns were linked by intermolecular hydrogen bonds CH•••O and Br•••O halogen bond with the distance of 2.968 Å and 3.527 Å.

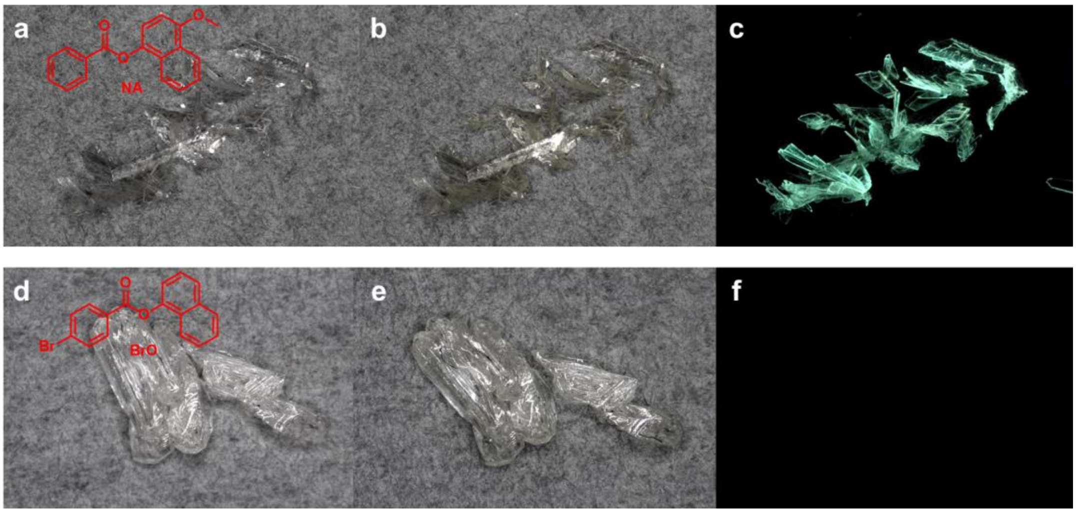

Figure S24. Photographs of single crystals of (a-c) **NA** and (d-f) **BrO** taken under ambient light, 365 nm excitation and fluorescence, respectively.

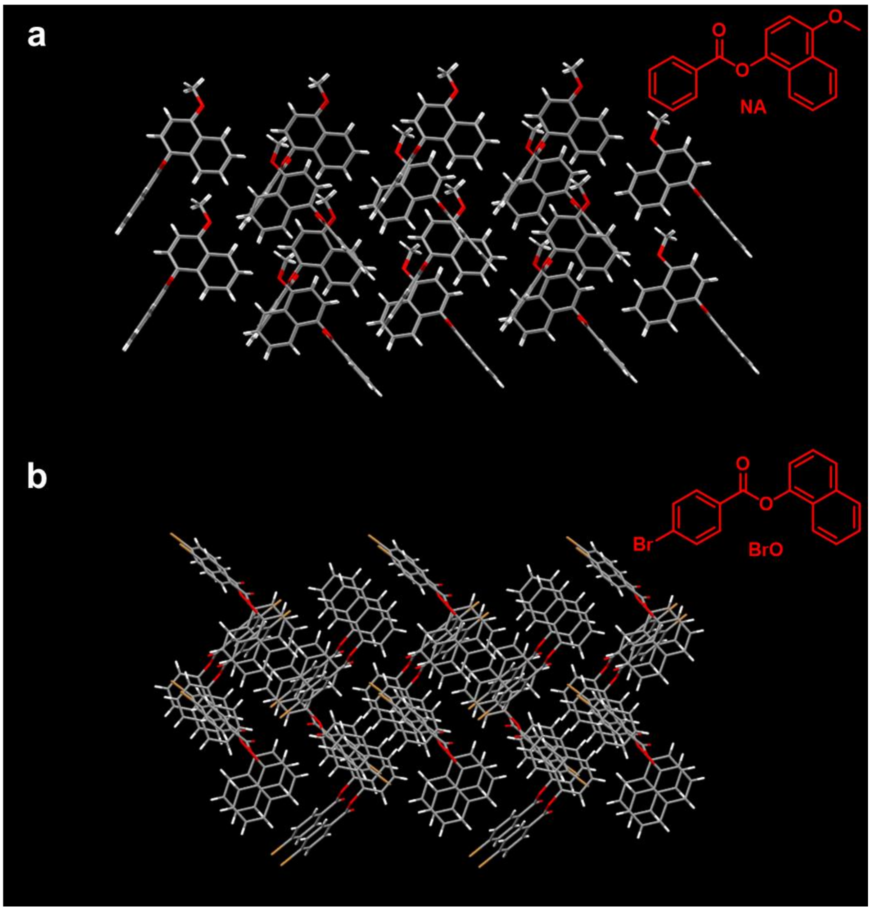

Figure S25. Molecular packing of (a) **NA** crystals and (b) **BrO** crystals.

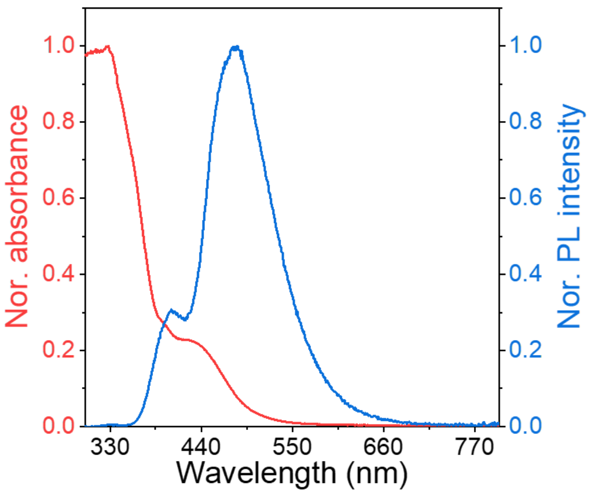

Figure S26. The spectral overlap region between PL (blue line) and absorption bands (red line) of **NPBr** is approximately 125 nm.

3. ^{1}H, ^{13}C NMR and High-resolution MS spectra

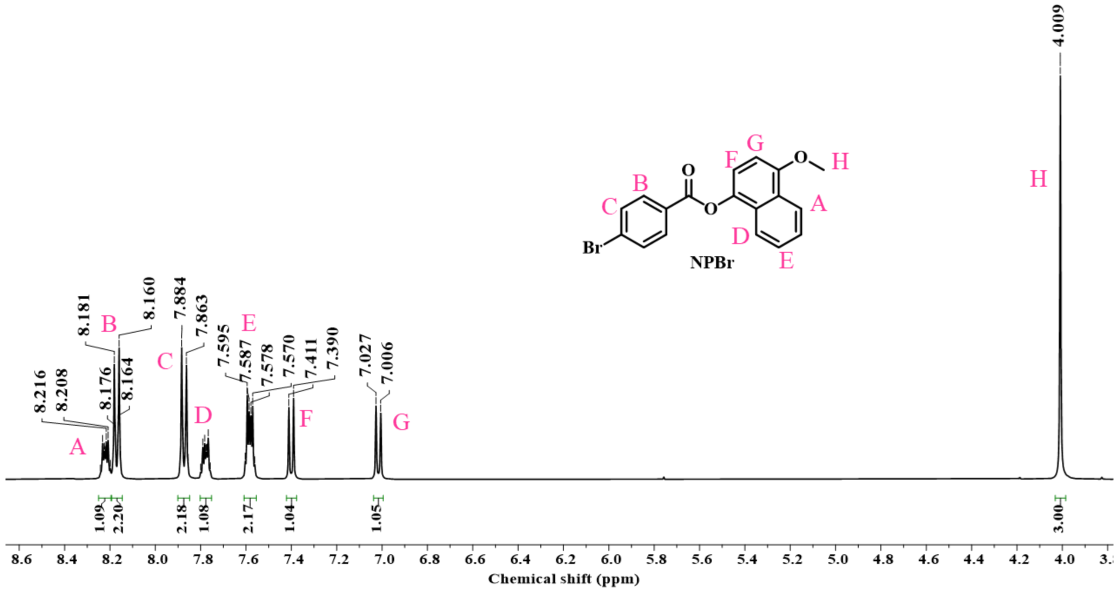

Figure S27. ^{1}H-NMR spectra of **NPBr**.

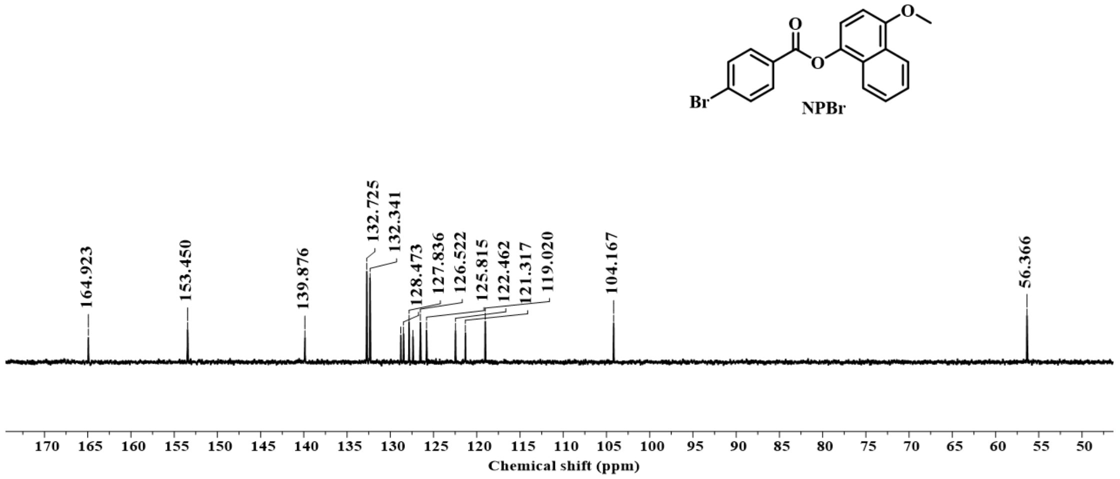

Figure S28. ^{13}C-NMR spectra of **NPBr**.

Formula (M)	Ion Formula	Calc m/z	m/z	Diff (ppm)
$C_{18}H_{13}O_3Br$	[M+H]	357.0121	357.0112	2.52

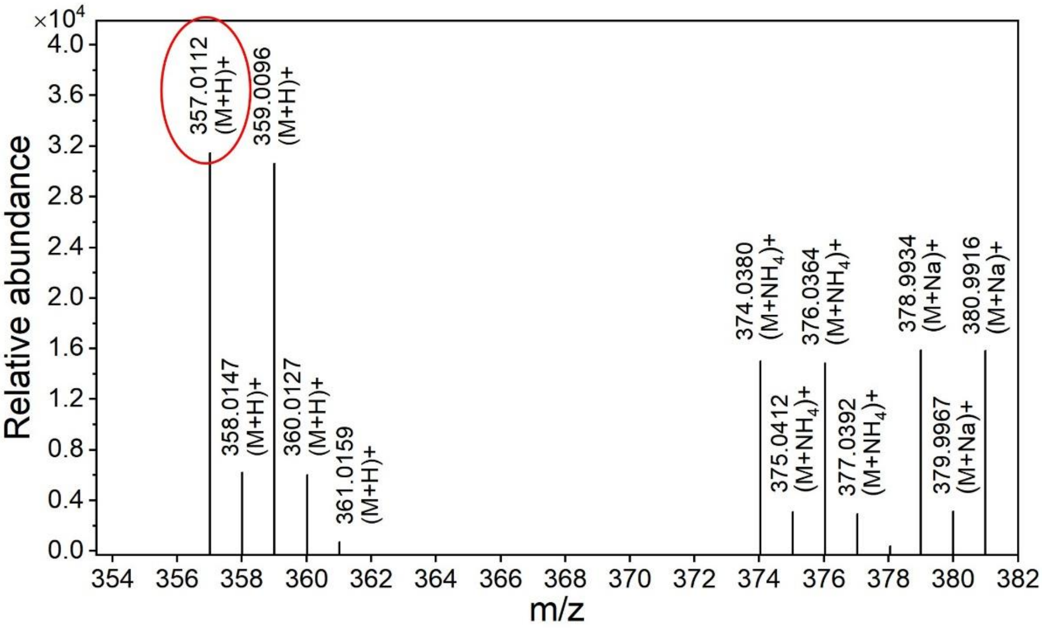

Figure S29. High-resolution MS spectra of **NPBr**.

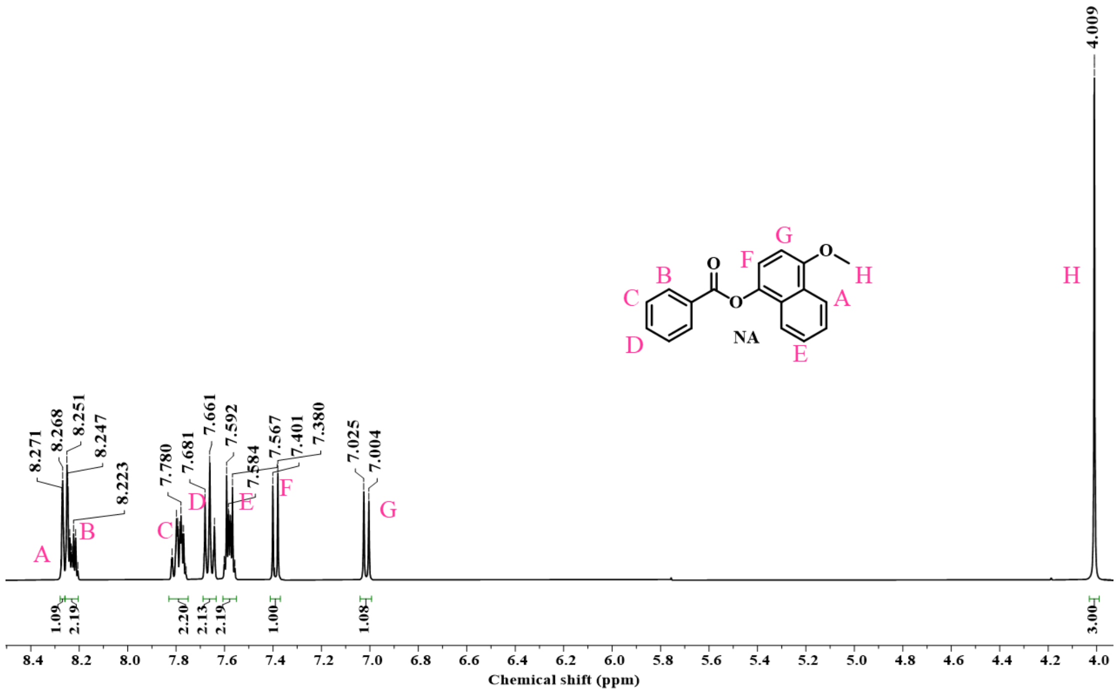

Figure S30. ^{1}H-NMR spectra of **NA**.

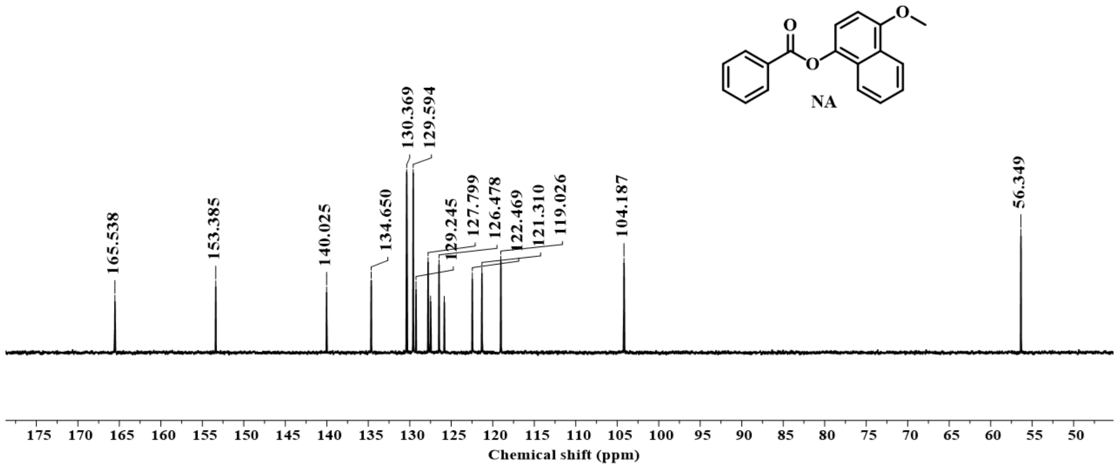

Figure S31. ^{13}C-NMR spectra of **NA**.

Formula (M)	Ion Formula	Calc m/z	m/z	Diff (ppm)
$C_{18}H_{14}O_3$	[M+H]	279.1016	279.1014	0.55

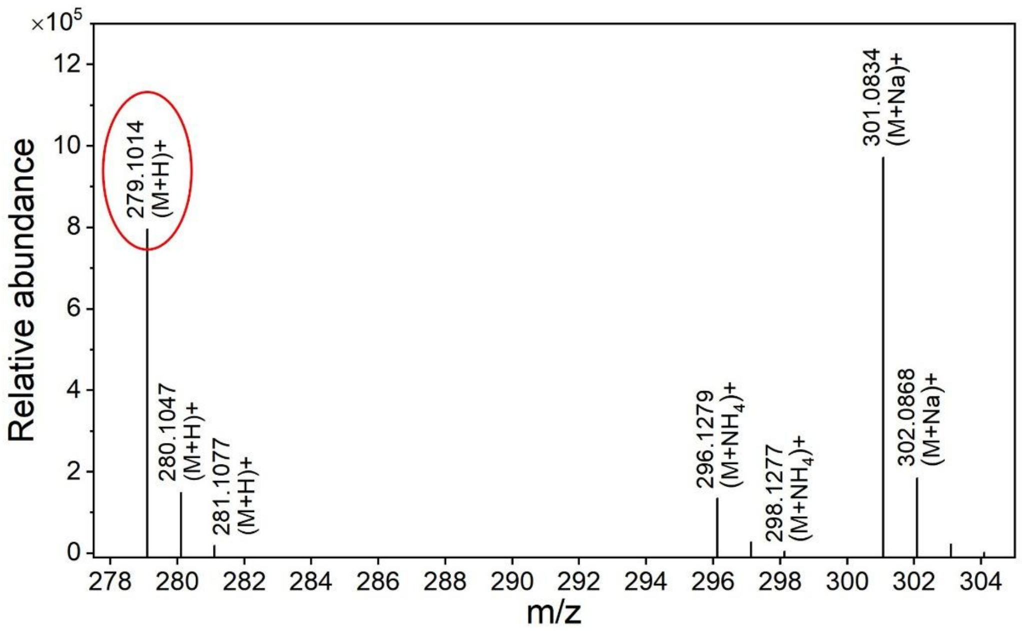

Figure S32. High-resolution MS spectra of **NA**.

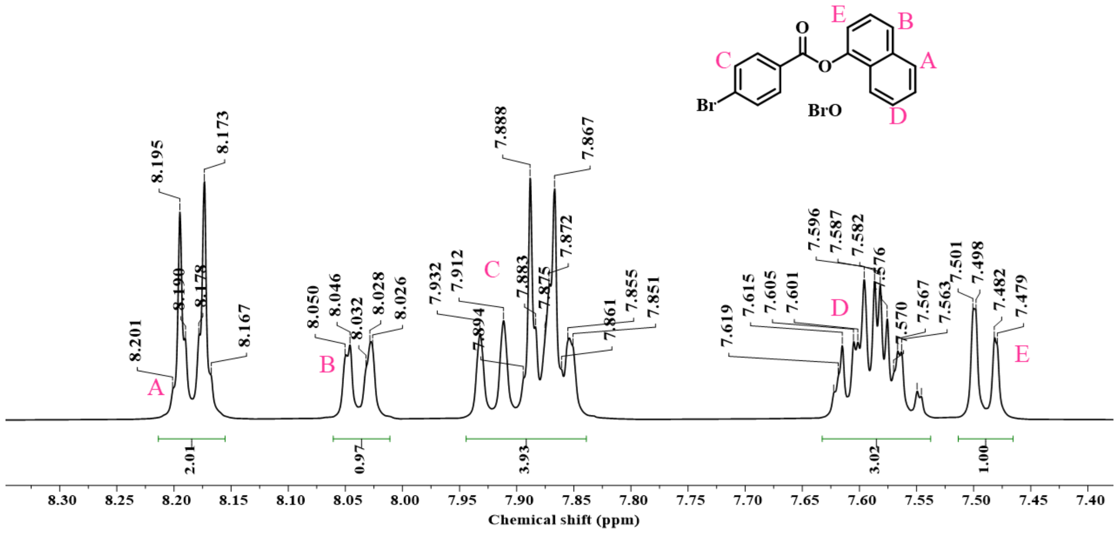

Figure S33. ^{1}H-NMR spectra of **BrO**.

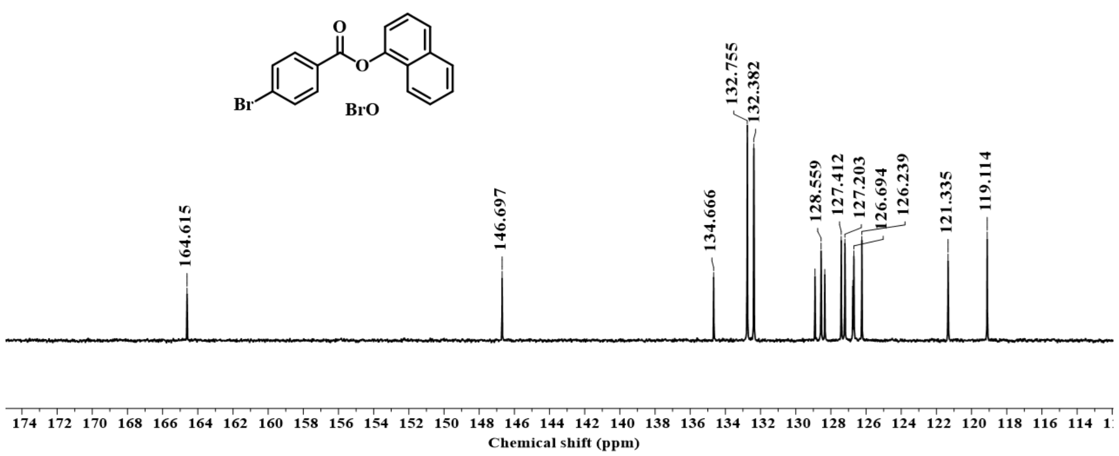

Figure S34. ^{13}C-NMR spectra of **BrO**.

Formula (M)	Ion Formula	Calc m/z	m/z	Diff (ppm)
$C_{17}H_{11}O_2Br$	[M+H]	327.0015	327.0013	0.67

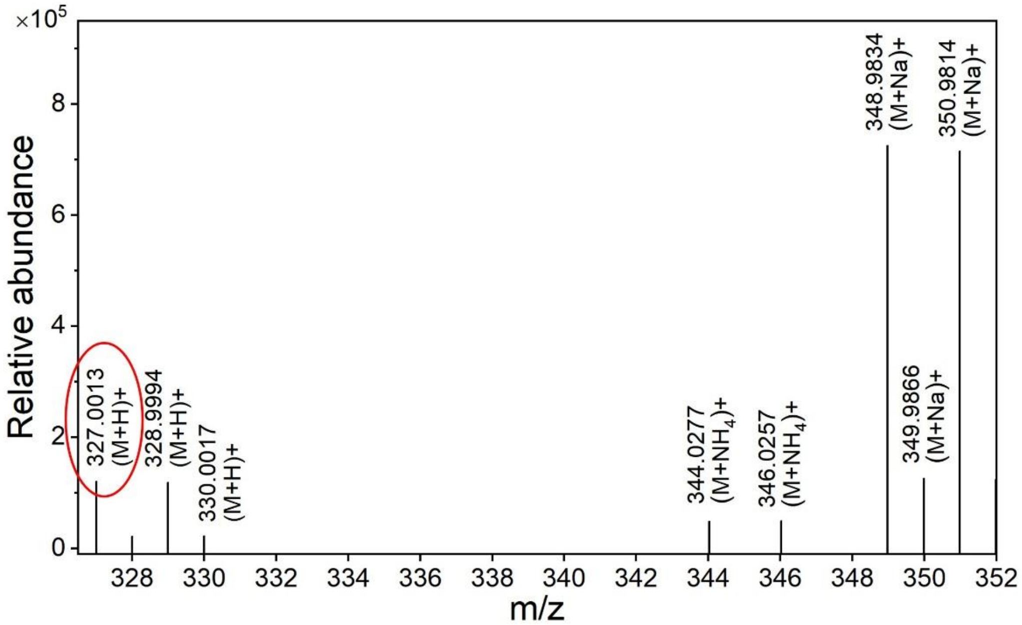

Figure S35. High-resolution MS spectra of **BrO**.

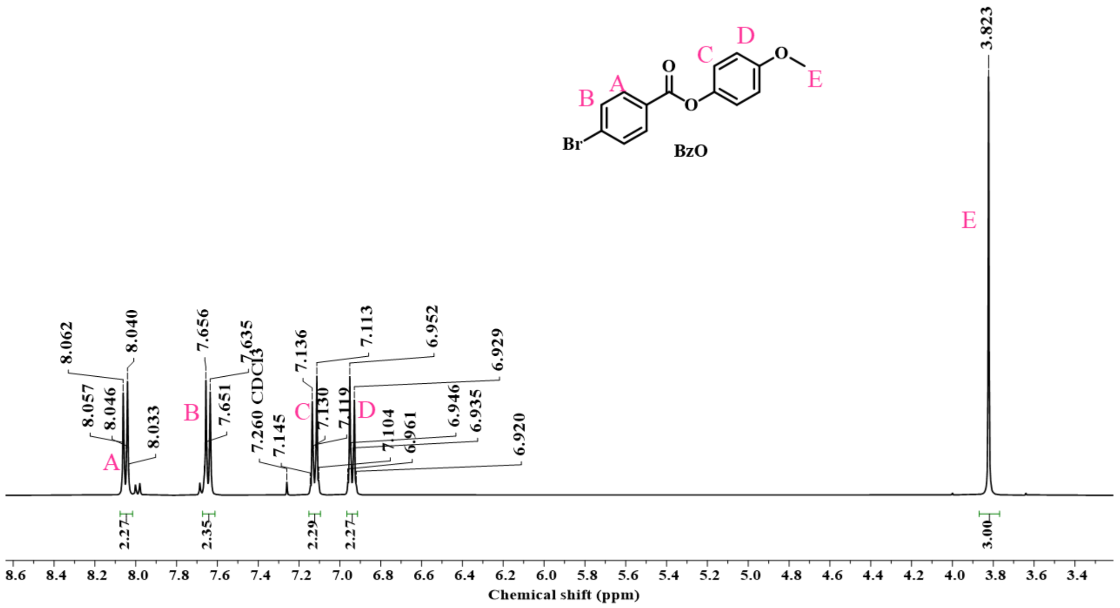

Figure S36. ^{1}H-NMR spectra of **BzO**.

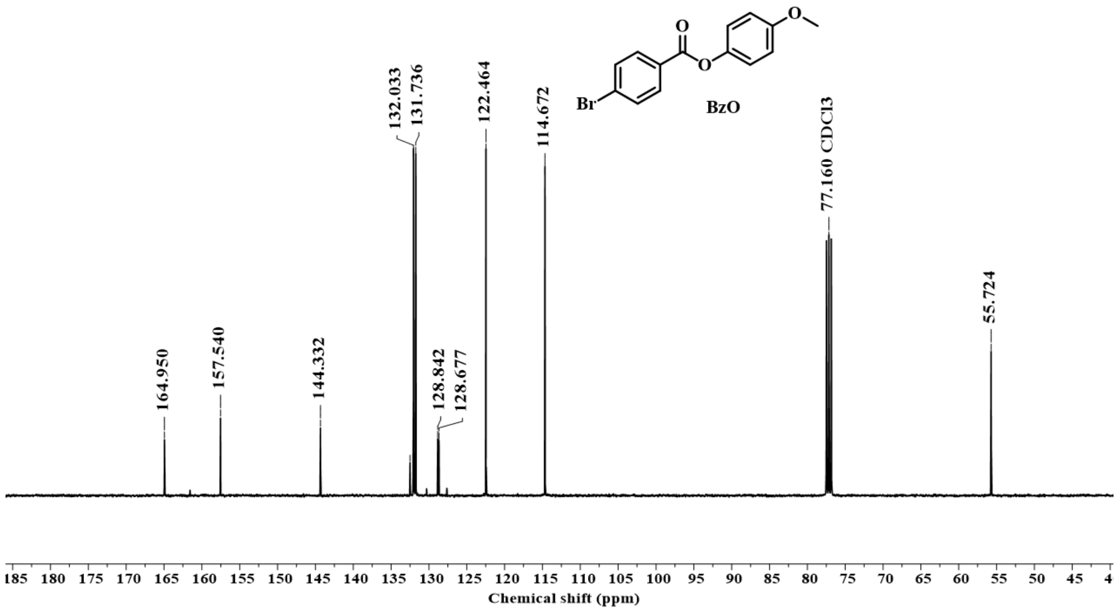

Figure S37. ^{13}C-NMR spectra of **BzO**.

Formula (M)	Ion Formula	Calc m/z	m/z	Diff (ppm)
$C_{14}H_{11}O_3Br$	[M+H]	306.9964	306.9968	-1.06

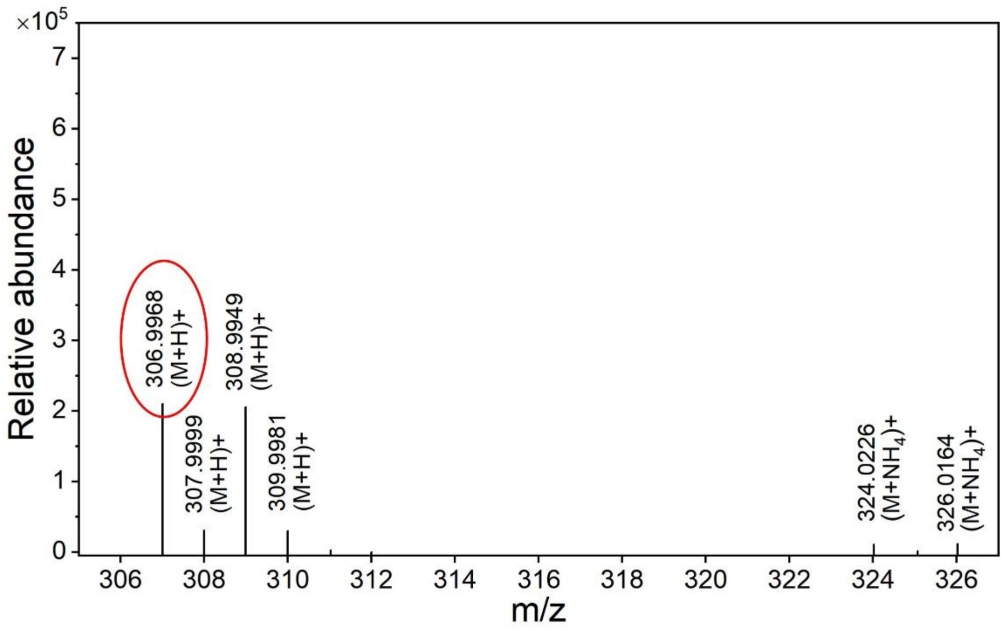

Figure S38. High-resolution MS spectra of **BzO**.

Table S1. Crystallographic data and structure refinement of **NPBr**, **NA** and **BrO** crystals.

Sample (CCDC NO.)	**NPBr (2422395)**	**NA (2422396)**	**BrO (2422397)**
Formula	$C_{18}H_{13}O_3Br$	$C_{18}H_{14}O_3$	$C_{17}H_{11}O_2Br$
Mr	357.19	278.29	327.17
Temperature (K)	150	306	280
Crystal system	*Monoclinic*	*Monoclinic*	*Monoclinic*
Space group	$P2_1/n$	Cc	$P2_1/c$
a (Å)	11.3851(4)	9.968(2)	9.3946(15)
b (Å)	4.6816(10)	35.569(5)	10.752(3)
c (Å)	27.3241(9)	9.5070(19)	14.608(4)
α (°)	90	90	90
β (°)	94.946(10)	121.615(8)	104.162(19)
γ (°)	90	90	90
V(Å^3)	1450.96(8)	2870.5(10)	1430.7(5)
Z	4	8	4
ρ_{calc} (g/cm^3)	1.635	1.288	1.519
2-Theta Range (°)	4.008 to 52.738	4.968 to 136.79	9.71 to 136.49
F (000)	720	1168	656.0
h, k, l_{max}	14,5,34	12,34,11	11,12,17
Reflections collected	10493	18658	13968
Independent reflections	2937[R_{int} = 0.0512]	5235[R_{int} = 0.1018]	2565[R_{int} = 0.0495]
Goodness-of-fit on F^2	1.036	1.051	1.107
Final R indexes [I>= 2σ (I)]	R_1 = 0.0355 wR_2 = 0.0807	R_1 = 0.0456 wR_2 = 0.1102	R_1 = 0.0369 wR_2 = 0.1023
Final R indexes [all data]	R_1 = 0.0541 wR_2 = 0.0897	R_1 = 0.0642 wR_2 = 0.1216	R_1 = 0.0430 wR_2 = 0.1076
Largest diff peak/hole/ e Å^{-3}	0.57/-0.50	0.22/-0.26	0.47/-0.58

$R_1 = \Sigma||F_o| - |F_c||/\Sigma|F_o|$, $wR_2 = [\Sigma w(F_o^2 - F_c^2)^2/\Sigma w(F_o^2)^2]^{1/2}$

Table S2. Crystallographic data and structure refinement of **CONP** crystal.

Sample (CCDC NO.)	**CONP (2484348)**
Formula	$C_{18}H_{13}BrO_3$
Mr	357.19
Temperature (K)	200
Crystal system	*Triclinic*
Space group	$P\bar{1}$
a (Å)	8.4195(8)
b (Å)	8.8783(9)
c (Å)	10.3568(10)
α (°)	73.825(2)
β (°)	87.590(2)
γ (°)	82.554(2)
V(Å^3)	737.25(12)
Z	2
ρ_{calc} (g/cm^3)	1.609
2-Theta Range (°)	10.454 to 144.706
F (000)	360.0
h, k, l_{max}	10,10,12
Reflections collected	11696
Independent reflections	2555[R_{int} = 0.0337]
Goodness-of-fit on F^2	1.167
Final *R* indexes [I>= 2σ (I)]	R_1 = 0.0409 wR_2 = 0.1050
Final *R* indexes [all data]	R_1 = 0.0411 wR_2 = 0.1051
Largest diff peak/hole/ e Å^{-3}	0.70/-0.56

$R_1 = \Sigma||F_o| - |F_c||/\Sigma|F_o|$, $wR_2 = [\Sigma w(F_o^2 - F_c^2)^2/\Sigma w(F_o^2)^2]^{1/2}$
